# Integrated Phytochemical, Network Pharmacology, and Molecular Docking Analyses of Triphala Extract Reveal Protective Effects Against H_2_O_2_-Induced Oxidative Hemolysis in G6PD-Deficient Erythrocytes

**DOI:** 10.3390/life16081359

**Published:** 2026-08-19

**Authors:** Aman Tedasen, Siriwimon Ranjuanjit, Nattacha Srirod, Kingkan Bunluepuech, Maria de Lourdes Pereira, Veeranoot Nissapatorn, Chutima Rattanawan, Naunpun Sangphech, Rachasak Boonhok, Orawan Sarakul

**Affiliations:** 1 Department of Medical Technology, School of Allied Health Sciences, Walailak University, Nakhon Si Thammarat 80160, Thailand; aman.te@wu.ac.th (A.T.); siriwimon.ra@mail.wu.ac.th (S.R.); nattacha.sr@mail.wu.ac.th (N.S.); kingkan.bu@wu.ac.th (K.B.); rachasak.bo@mail.wu.ac.th (R.B.); 2 Research Excellence Center for Innovation and Health Products (RECIHP), Walailak University, Nakhon Si Thammarat 80160, Thailand; nissapat@gmail.com; 3 Department of Medical Sciences, CICECO-Aveiro Institute of Materials, University of Aveiro, 3810-193 Aveiro, Portugal; mlourdespereira@ua.pt; 4 Futuristic Science Research Center, School of Science, World Union for Herbal Drug Discovery (WUHeDD), Walailak University, Nakhon Si Thammarat 80160, Thailand; 5 Department of Medical Science, Amnatcharoen Campus, Mahidol University, Mueang Amnat Charoen 37000, Thailand; chutima.ran@mahidol.ac.th; 6 Department of Medical Technology, Faculty of Allied Health Sciences, Thammasat University, Khlong Luang, Pathumthani 12120, Thailand; naunpuns@tu.ac.th; 7 Hematology and Transfusion Sceince Research Center (HTSRC), Walailak University, Nakhon Si Thammarat 80160, Thailand

**Keywords:** triphala, G6PD deficiency, hemolysis, oxidative stress, phytochemical profiling, network pharmacology, molecular docking

## Abstract

**Background/Objectives**: Oxidative stress is a major cause of RBC membrane damage, especially in individuals with G6PD deficiency who have impaired antioxidant defenses. Triphala, a phenolic-rich herbal formulation with known antioxidant activity, was evaluated for its phytochemical profile, antioxidant and anti-hemolytic effects, and molecular mechanisms in H_2_O_2_-induced oxidative stress models using normal and G6PD-deficient human RBCs. **Methods**: Triphala aqueous extract was characterized using LC-MS and GC-MS. Antioxidant activity was evaluated by DPPH and ABTS assays. Cytotoxicity, membrane stability, and protection against H_2_O_2_-induced hemolysis were assessed in normal and G6PD-deficient RBCs. Network pharmacology, molecular docking and MD simulation analyses were performed to predict antioxidant-related mechanisms. Statistical analysis was conducted using one-way ANOVA (*p* < 0.05). **Results**: LC-MS identified gallic acid as the predominant phenolic compound, while GC-MS revealed pyrogallol as the major constituent. The extract showed strong radical scavenging activity and significantly reduced H_2_O_2_-induced hemolysis in both normal and G6PD-deficient RBCs without cytotoxicity (*p* < 0.05). Network pharmacology revealed that G6PD-related antioxidant regulation, oxidative stress response, and inflammatory signaling pathways are the key enriched biological processes. Network pharmacology analysis ranked PPARG, PTGS2, EGFR, MMP9, TLR4, ACE, REN, PPARA, SERPINE1, and MMP2 as the top hub proteins, highlighting their central roles in oxidative stress, inflammation, and metabolic signaling pathways. Kynurenic acid binds strongly to PTGS2 (COX-2) and ACE with binding affinities below −7.0 kcal/mol, forming multiple hydrogen bonds that stabilize its interactions within the active sites. MD simulations confirmed that kynurenic acid binds stably to ACE and PTGS2, with RMSD values plateauing near 2.4 Å and 3.0 Å, RMSF values mostly below 2 Å, and recurrent hydrogen bonding and electrostatic contacts with key residues, collectively underscoring its conformational stability, adaptive flexibility, and modulatory potential. **Conclusions**: Triphala aqueous extract exhibits potent antioxidant and anti-hemolytic activities and may serve as a natural adjunct strategy for reducing oxidative damage in G6PD deficiency and related RBC disorders.

## 1. Introduction

Glucose-6-phosphate dehydrogenase (G6PD) deficiency is an inherited enzymatic disorder of red blood cells caused by mutations in the G6PD gene, affecting an estimated 400 million people worldwide [1,2,3,4,5,6,7,8,9,10,11,12]. G6PD deficiency is the most common enzymatic disorder of red blood cells, characterized by impaired production of NADPH, which is essential for maintaining redox balance through the reduction of oxidized glutathione [13]. Because mature erythrocytes lack nuclei and mitochondria, they cannot synthesize new enzymes and rely exclusively on the pentose phosphate pathway, mediated by G6PD, to generate NADPH and protect against oxidative damage. In the absence of sufficient G6PD activity, red blood cells become highly susceptible to oxidative stress and undergo premature destruction, leading to hemolytic anemia [14]. This X-linked genetic disorder compromises cellular defense against oxidative stress and manifests clinically during oxidative challenges such as infections, ingestion of fava beans, or exposure to certain drugs, including antimalarials and sulfonamides [15]. Consequences may include acute hemolytic episodes, neonatal hyperbilirubinemia, and in severe cases, kernicterus if diagnosis or management is delayed. Given its prevalence and clinical impact, identifying safe and effective antioxidant interventions is critical to mitigate hemolytic risk and improve outcomes in affected individuals [16]. The mechanism of hemolysis in G6PD deficiency involves impaired NADPH generation, which prevents glutathione from remaining in its reduced, protective state, thereby allowing reactive oxygen species (ROS) to accumulate and damage hemoglobin, leading to the formation of Heinz bodies. As these abnormal red blood cells pass through the spleen, macrophages remove the Heinz bodies, producing characteristic bite and blister cells, or destroying the fragile cells entirely, resulting in hemolysis [17]. Clinical manifestations include anemia with fatigue, pallor, and tachycardia, jaundice and dark urine due to excess bilirubin, and neonatal hyperbilirubinemia that may progress to kernicterus if untreated. Diagnosis is confirmed by measuring G6PD enzyme activity, and management focuses on avoiding oxidative triggers, treating infections promptly, providing supportive care such as hydration, and administering blood transfusions in severe cases [1]. Therefore, preventing hemolytic episodes by managing oxidative stress is vital in G6PD deficiency. Alongside standard clinical care and trigger avoidance, natural antioxidant-rich products may help enhance redox balance, stabilize membranes, and protect red blood cells from oxidative damage.

Triphala, an ancient Ayurvedic formulation composed of equal parts of Haritaki (*Terminalia chebula*), Bibhitaki (*Terminalia bellirica*), and Amalaki (*Phyllanthus emblica*), is widely recognized for its robust antioxidant activity [18]. Its therapeutic potential arises from a diverse profile of polyphenols, flavonoids, and organic acids, with key constituents such as gallic acid, which scavenges free radicals and reduces DNA damage; ellagic acid, a polyphenol that mitigates oxidative stress and inflammation; and ascorbic acid (vitamin C), abundant in Amla, which neutralizes reactive oxygen species (ROS) [19]. Triphala exhibits robust antioxidant activity driven by its rich vitamin C, polyphenol, and flavonoid content, effectively neutralizing free radicals, reducing lipid peroxidation, preventing DNA and cellular damage, and boosting endogenous antioxidant enzymes, such as SOD, CAT, and GSH, across various in vitro and in vivo models [20]. Triphala exhibits strong antioxidant potential by scavenging free radicals, restoring both enzymatic and non-enzymatic antioxidant defenses, and reducing lipid peroxidation. Beyond its antioxidant role, it is recognized for chemopreventive, chemotherapeutic, immunomodulatory, and radioprotective properties. Accumulating evidence shows that Triphala modulates diverse cell signaling pathways, including ERK, MAPK, NF-κB, Akt, c-Myc, VEGFR, mTOR, tubulin, p53, cyclin D1, and regulators of apoptosis, underscoring its multifaceted biological activity [18]. Triphala stimulates the production of red blood cells and hemoglobin, making it potentially beneficial for managing anemia and improving overall blood health [21]. Although Triphala is well-established as a potent antioxidant with documented benefits in cancer, inflammation, and metabolic disorders, its specific role in protecting red blood cells from oxidative stress and preventing hemolysis has not yet been clearly reported. Given that G6PD-deficient erythrocytes are highly vulnerable to oxidative damage, further investigation into Triphala’s polyphenols, flavonoids, and organic acids may reveal whether these natural compounds can enhance redox balance, stabilize membrane integrity, and reduce susceptibility to hemolytic episodes. Such studies would provide valuable insight into the potential of Triphala as a complementary antioxidant intervention for red blood cell protection. Due to its antioxidant, anti-inflammatory, immunomodulatory, antimicrobial, anti-cancer, and cytoprotective properties, it has been utilized for centuries in traditional medicine and has garnered significant scientific attention [22,23,24,25,26]. In addition to gallic acid, their phenolic constituents, including ellagic acid, chebulagic acid, chebulinic acid, corilagin, and other hydrolyzable tannins, are largely responsible for these biological properties owing to their potent free radical-scavenging and metal-chelating activities. Additionally, compared to individual phytochemicals alone, the combination of three medicinal fruits offers a wide range of bioactive phytochemicals that may have complementary or synergistic antioxidant effects, increasing its therapeutic potential. Triphala was selected for the present study because it is one of the most extensively investigated and chemically characterized polyherbal formulations, with substantial evidence supporting its antioxidant efficacy and favorable safety profile. Unlike many antioxidant-rich herbal preparations that rely primarily on a single botanical source, Triphala contains multiple classes of phenolic compounds that target different oxidative stress pathways simultaneously [22,26,27,28,29]. This phytochemical diversity provides a strong mechanistic rationale for evaluating its protective effects against oxidative damage in G6PD-deficient erythrocytes.

AI-driven computational methodologies, such as drug-likeness evaluation, ADMET profiling (Absorption, Distribution, Metabolism, Excretion, and Toxicity), network pharmacology, and molecular docking, form the foundation of modern in silico drug discovery. These approaches provide systematic strategies to assess phytoconstituents for pharmacokinetic behavior, drug-likeness, and multi-target interactions, while enabling researchers to rapidly filter large libraries of plant-derived compounds to predict their therapeutic viability [30]. By identifying bioactive molecules, mapping their molecular targets, and elucidating pathways involved in oxidative stress and red blood cell stability, these computational tools streamline the discovery process and reduce reliance on costly, time-consuming in vitro and in vivo studies, thereby accelerating the development of novel natural product-based therapeutics [31]. These approaches enable the identification of bioactive compounds, their molecular targets, and pathways implicated in oxidative stress and red blood cell (RBC) stability. Network pharmacology, introduced by Hopkins in 2007, revolutionized drug discovery by shifting from the traditional one-drug–one-target paradigm to a systems-level, multi-target perspective, integrating omics technologies, systems biology, bioinformatics, computational modeling, and artificial intelligence to construct multilayered drug–target–disease networks that capture the complexity of molecular interactions [32]. Such networks accelerate target identification, support drug repurposing, and uncover synergistic mechanisms of multi-component or polyherbal therapies, as demonstrated in diverse therapeutic areas including cancer, ischemic stroke, and metabolic disorders [33]. Unlike conventional pharmacology’s narrow focus on single targets, this holistic approach maps phytochemicals to their targets, pathways, and clinical phenotypes, offering deeper insight into disease pathogenesis and treatment [34].

Therefore, comprehensive phytochemical characterization and functional validation in oxidative hemolysis models are required. In this context, the present study aimed to (i) characterize the phytochemical composition of Triphala aqueous extract and evaluate its antioxidant capacity; (ii) assess its cytotoxicity in human RBCs and investigate its protective effect against H_2_O_2_-induced hemolysis in both healthy and G6PD-deficient erythrocytes, with the objective of exploring its potential as a natural adjunct strategy for mitigating oxidative damage in G6PD-related RBC disorders; and (iii) integrate in silico approaches, including ADMET prediction, network pharmacology, and molecular docking, to elucidate the molecular mechanisms underlying the antioxidant and anti-hemolytic effects of Triphala extract in G6PD-deficient RBCs and evaluate its potential as a therapeutic candidate for oxidative stress–related hematological disorders.

## 2. Materials and Methods

### 2.1. Plant Material and Aqueous Decoction

Triphala extract was prepared by mixing the 3 dried fruits (Haritaki, Bibhitaki, and Amalaki) in equal parts. A total of 100 g of the fruit was mixed with 1000 mL of distilled water and boiled for 2 h while stirring constantly. The mixture was filtered and then freeze-dried to get the crude aqueous extract.

### 2.2. LC-MS Analysis

LC-MS analysis was conducted at the Proteomic Service Center, Faculty of Medical Technology, Mahidol University (Nakhon Pathom, Thailand). Triphala extract (1 mg/mL) was prepared in 0.1% formic acid, vortexed, centrifuged, and filtered (0.22 µm) prior to analysis. Chromatographic separation was performed on an HPLC system (Bruker, Bremen, Germany) with a C18 column (5 µm, 4.6 mm × 150 mm; Phenomenex Inc., Torrance, CA, USA), using water containing (0.1% formic acid, A) and acetonitrile (0.1% formic acid, B) under gradient elution. An injection of 10 µL was introduced into the system at a flow rate of 0.5 mL/min. The ultrahigh definition accurate Q-TOF mass spectrometer (Bruker Daltonics, Bremen, Germany). Mass spectrometry was carried out using an ESI-QTOF instrument (Bruker, Germany) in both positive and negative electrospray ionization (ESI) modes over an *m*/*z* range of 50–1000 Data. Data processing was performed using MetaboScape^®^ (Bruker Daltonics). Metabolites were identified by comparison with MetaboBase and MassBank (MONA) databases. Metabolites were examined using positive electrospray ionization (ESI+) mode for LC-MS analysis. Protonated molecular ions ([M + H]^+^), computed molecular masses, isotope distribution, chromatographic retention behavior, and database matching were used to tentatively assign candidate compounds. The final metabolite list contained only compounds with library matching scores of at least 90%. To increase annotation confidence, mass accuracy (ppm) and chromatographic retention characteristics were taken into account when available. Peak intensity from the total ion chromatogram (TIC) was used to estimate relative abundance.

### 2.3. GC-MS Analysis

Triphala extract was prepared for component analysis by GC-MS. The extract was dissolved in methanol, followed by centrifugation at 12,000 rpm. The clear solution was subjected to GC-MS. GC-MS analysis using an Agilent system consisting of a GC 6890 along with a 5973-mass selective detector (MSD). Chromatographic separation was achieved on a ZB-5MS Plus capillary column(Phenomenex Inc., Torrance, CA, USA) (30 m × 0.25 mm, 0.25 μm film thickness). The injection was performed in split mode (1:5) at 280 °C. Helium was used as carrier gas at a constant flow rate of 1 mL/min. The temperature program was set as follows: initial temperature of 60 °C, followed by a ramp to 280 °C at a rate of 5 °C/min, with a final hold of 10 min, resulting in a total run time of 54 min. Mass spectrometric detection was carried out under electron ionization (EI) mode, with a mass scan range of 35–550 amu. Compound identification was analyzed by comparing the obtained mass spectra with the W10N14.L database. Compound annotation was based on spectral similarity scores, retention time, and molecular weight. Only compounds with spectral matching scores greater than 80% were considered high-confidence identifications and included in the final analysis. Relative abundance was expressed as normalized peak area (%) of the total ion chromatogram.

### 2.4. Antioxidant Assays

#### 2.4.1. DPPH Radical Scavenging Assay

The scavenging activity against the 2,2-diphenyl-1-picrylhydrazyl (DPPH) radical was measured following established protocols [27]. Fifty μL of each extract dilution, 0.25 to 4 mg/mL was mixed with 150 μL of 0.2 mM DPPH solution (in methanol). The mixture was incubated for 30 min in the dark, and the absorbance was detected at 517 nm. The radical scavenging activity was calculated using the formula:

Radical Scavenging Activity (%) = (A_control_ − A_sample_)/A_control_ × 100

#### 2.4.2. ABTS Radical Cation Decolorization Assay

The ABTS radical cation assay was performed using a modified method described previously [35]. The 2,2′-azino-bis (3-ethylbenzothiazoline-6-sulfonic acid) (ABTS) radical cation was generated by reacting ABTS stock solution with 2.45 mM potassium persulfate (K_2_S_2_O_8_). The mixture was allowed to stand in the dark for 12–16 h. The ABTS working solution was prepared by diluting the stock with PBS until the absorbance was 0.70–0.02 at 734 nm. Extract solutions (0.25 to 4 mg/mL) were mixed with the ABTS working solution, and the decrease in absorbance was measured after 6 min. Results were calculated using the same formula as the DPPH assay, and the IC_50_ value was determined. Scavenging activity and IC_50_ values were calculated as described for the DPPH assay.

### 2.5. Blood Sample Collection

Blood samples (6 mL per participant) were collected from three healthy male volunteers (control group) and three male volunteers with G6PD deficiency. Participants were age between 18–35 years who had no known history of acute illness, hematological disorders other than G6PD deficiency, or current use of medications that could influence erythrocyte integrity. Blood was collected into ethylenediaminetetraacetic acid (EDTA) tubes and divided for complete blood count (CBC) analysis and G6PD deficiency screening using the fluorescence spot test (FST). Healthy volunteers exhibited normal hemoglobin concentrations (13.3 ± 0.5 g/dL), red blood cell indices (MCV = 84.5 ± 2.4 fL, MCH = 27.97 ± 0.90 pg, MCHC = 33.27 ± 0.25 g/dL), and normal G6PD screening results. G6PD-deficient participants had normal hemoglobin concentrations (13.8 ± 1.7 g/dL) and red blood cell indices (MCV = 85.1 ± 4.8 fL, MCH = 28.47 ± 1.03 pg, MCHC = 33.17 ± 0.29 g/dL), but showed deficient G6PD status as determined by the FST. Four milliliters of EDTA-anticoagulated blood from each participant were used for the hemolysis and anti-hemolysis assays. The study was conducted in accordance with ethical guidelines and received formal approval from the Human Research Ethics Committee, Walailak University (Certificate No. WUEC-23-031-01). All participants were informed on the study objectives and signed informed consent before sample collection.

### 2.6. Erythrocyte Preparation and Evaluation of Protective Effects Against Oxidative Hemolysis

Blood samples were centrifuged at 3000 rpm for 10 min to remove plasma and buffy coat. RBCs were washed 3 times with phosphate-buffered saline (PBS, pH 7.4), and a 10% suspension was prepared for analysis.

#### 2.6.1. Hemolysis Assay in Normal and G6PD-Deficient Human RBCs

The 10%RBC suspension of normal and G6PD deficiency were incubated with Triphala extract or ascorbic acid in approximately the same concentration for 4 h at 37 °C in a water bath. RBC incubated in distilled water was used as the positive control (PC) (100% hemolysis), and PBS-treated RBC was included as negative control (NC). All tubes were centrifuged for 10 min at 2000 rpm. The supernatant was collected for hemolysis determination by detecting the absorbance (Abs) at 540 nm [36].

Hemolysis (%) were calculated using the following formula:

Hemolysis (%) = [(Abs_Treat_ − Abs_NC_)/Abs_PC_] × 100

#### 2.6.2. Anti-Hemolysis Assay in H_2_O_2_-Induced Condition

Oxidative hemolysis was induced using Hydrogen Peroxide (H_2_O_2_) on previously validated erythrocyte protection assays. RBC suspension was pre-incubated with various concentrations of the Triphala extract (25 to 500 g/mL) for 30 min at 37 °C. Following pre-incubation, H_2_O_2_ was added to achieve a final concentration of 10 mM. The samples were then incubated at 37 °C for 3 h. The relative hemolysis was calculated in comparison with the hemolysis in the H_2_O_2_ treated (positive control), representing 100% hemolysis. For negative control (0% hemolysis) phosphate buffer saline was used. After incubation, the samples were centrifuged, and the absorbance of the supernatant (containing the released hemoglobin) was measured at 540 nm and the percentage of hemolysis was calculated.

### 2.7. Prediction of Chemo-Informatics and Drug Likeness

The physicochemical and drug-likeness properties of compounds derived from Triphala extracts were systematically evaluated using the SwissADME web tool (http://www.swissadme.ch (accessed on 10 March 2026)) [37]. Key molecular descriptors, including molecular weight, partition coefficient (LogP), topological polar surface area (TPSA), hydrogen bond donors (HBD), hydrogen bond acceptors (HBA), and rotatable bonds, were calculated to assess compliance with established drug-likeness filters such as Lipinski’s Rule of Five, Ghose, Veber, Egan, and Muegge criteria. These parameters provide critical insights into solubility, permeability, and oral bioavailability potential.

### 2.8. Pharmacokinetics and Toxicity (ADMET) Prediction

In addition to physicochemical and drug-likeness profiling, the pharmacokinetic behavior of Triphala extract compounds was systematically modeled using in silico ADMET predictions. The canonical SMILES notation of each compound was submitted to the pkCSM platform (www.biosig.lab.uq.edu.au/pkcsm/prediction (accessed on 10 March 2026)), which applies graph-based signatures to estimate absorption, distribution, metabolism, excretion, and toxicity parameters [38]. This approach enabled the evaluation of gastrointestinal absorption, blood–brain barrier (BBB) permeability, cytochrome P450 enzyme inhibition, and potential toxicological liabilities such as hepatotoxicity, mutagenicity, and hERG channel blockade.

### 2.9. Molecular Targets of Triphala-Derived Compounds in Antioxidant Defense Against RBC Hemolysis in H_2_O_2_-Induced Normal and G6PD-Deficient Models

The chemical structures of Triphala-derived compounds were retrieved from the PubChem database (https://pubchem.ncbi.nlm.nih.gov/ (accessed on 10 March 2026)) in canonical SMILES format. These SMILES strings were submitted to the Swiss Target Prediction platform (http://www.swisstargetprediction.ch (accessed on 10 March 2026)) to forecast potential human protein targets under default parameters [39]. To ensure consistency in gene/protein nomenclature, removal of duplicates, and harmonization of annotations, the predicted targets were subsequently standardized by mapping to UniProt entries (https://www.uniprot.org/ (accessed on 13 March 2026)). To establish disease relevance, a catalog of oxidative stress–associated genes was assembled by querying the GeneCards database (https://www.genecards.org/ (accessed on 13 March 2026)) with the terms “oxidative stress,” “hemolysis,” and “G6PD” [40]. The curated compound-specific targets were then compared against this disease gene set using the InteractiVenn (https://www.interactivenn.net/ (accessed on 26 March 2026)) [41]. The overlapping entries identified through this integrative analysis represent a refined panel of candidate molecular targets that may mediate the antioxidant defense of Triphala compounds against RBC hemolysis in both H_2_O_2_-induced normal and G6PD-deficient models. This systematic approach provides a robust framework for linking phytochemical constituents to disease-associated pathways, thereby supporting mechanistic insights into their therapeutic potential.

### 2.10. Protein-Protein Interaction (PPI) Network Construction

To investigate the functional relationships among the shared molecular targets of Triphala-derived bioactive compounds and disease-associated genes, a protein–protein interaction (PPI) network of *Homo sapiens* was constructed using STRING version 12.0 (http://string-db.org (accessed on 15 June 2026)) with a confidence score threshold of 0.40 to retain medium-confidence associations [42]. The resulting dataset, consisting of proteins as nodes and predicted associations as edges, was exported in TSV and PNG formats and imported into Cytoscape version 3.10.4 (https://cytoscape.org (accessed on 15 June 2026)) for visualization and topological analysis.

Network properties, including node degree, clustering coefficient, and betweenness centrality, were quantified using the Network Analyzer tool to characterize overall network architecture. To identify key regulatory proteins, CytoHubba version 0.1 was applied to rank nodes based on degree centrality, and the top ten nodes were designated as hub targets. These hub proteins represent potential critical regulators within the network and were prioritized for further functional interpretation and experimental validation [43].

### 2.11. Gene Ontology (GO) and Kyoto Encyclopedia of Genes and Genomes (KEGG) Enrichment Analysis

To systematically investigate the biological significance of the shared targets between Triphala-derived bioactive compounds and disease-associated genes, Gene Ontology (GO) and Kyoto Encyclopedia of Genes and Genomes (KEGG) enrichment analyses were performed using ShinyGO version 0.85 (http://bioinformatics.sdstate.edu/go/ (accessed on 15 June 2026)) [44]. These analyses were conducted to elucidate the functional roles and signaling pathways associated with overlapping targets in *H. sapiens*. Statistical significance was determined using a false discovery rate (FDR) threshold of ≤0.05. From a pool of up to 2000 candidate terms, the top 20 enriched categories were selected for both GO and KEGG analyses to ensure focus on the most biologically relevant results. For GO enrichment, results were organized into the three principal domains: Biological Process (BP), Cellular Component (CC), and Molecular Function (MF). Enriched terms were visualized as plots ranked by −log10(FDR), enabling comparative assessment of how Triphala-derived compounds may influence cellular processes, subcellular localization, and molecular activities. In parallel, KEGG pathway enrichment was performed under identical statistical thresholds to identify key signaling cascades implicated in disease pathogenesis and potentially modulated by Triphala bioactive compounds. Together, these enrichment analyses provide a comprehensive functional map of the shared targets, highlighting biological pathways that drive disease phenotypes and offering mechanistic insights into the therapeutic relevance of Triphala-derived compounds. By integrating GO and KEGG results, this study underscores the complex interplay among molecular functions, signaling networks, and disease mechanisms, thereby laying the groundwork for hypothesis generation and future experimental validation.

### 2.12. Computational Docking and Ligand Optimization

Molecular docking simulations were conducted using an integrated workflow of computational platforms, including UCSF Chimera (version 10.17.3), AutoDock Tools (ADT version 4.2.6), BIOVIA Discovery Studio (version 2025), the RCSB Protein Data Bank (https://www.rcsb.org/ (accessed on 20 June 2026)), and the PubChem database (https://pubchem.ncbi.nlm.nih.gov/ (accessed on 20 June 2026)). Three-dimensional structures of Triphala-derived bioactive compounds were retrieved from PubChem in SDF format and subjected to charge calculation, hydrogen bond optimization, and energy minimization in UCSF Chimera using the Steepest Descent (steps at 10,000, size at 0.02 angstrom) and Conjugate Gradient algorithms (Conjugate gradient steps at 10 and size at 0.02 angstrom). Structural optimization was performed under the AMBER force field with AM1-BCC charge assignment, and hydrogen atoms were added at physiological pH (7.4) to ensure accurate protonation states. Bond orders, torsional angles, and molecular topology were further refined in UCSF Chimera, after which the ligands were converted into AutoDock-compatible formats (PDB and PDBQT) using AutoDock Tools. For comparative docking validation, co-crystallized ligands from the target proteins were prepared using the same protocol and employed as positive controls. To ensure the reliability of the docking results, a re-docking procedure was performed in which co-crystallized ligands from the reference protein structures were docked back into their respective binding sites, and the predicted poses were compared with the experimentally determined crystallographic conformations. This validation step confirmed the robustness of the docking protocol and increased confidence in the accuracy of the predicted protein–ligand interactions. The standardized workflow ensured consistency across ligand preparation and docking simulations, thereby enhancing the reliability of binding predictions [45].

### 2.13. Protein Structure Preparation and Docking Analysis

Three-dimensional crystal structures of top 10 key disease-related proteins, including PPARG (7WOX), PTGS2 (5KIR), EGFR (7U9A), MMP9 (1GKC), TLR4 (3VQ2), ACE (1O86), REN (2V16), PPARA (7E5I), SERPINE1 (7AQF) and MMP2 (8H78), were retrieved from the RCSB Protein Data Bank, ensuring resolutions below 3.0 Å for structural accuracy. Non-essential water molecules and co-crystallized ligands were removed using BIOVIA Discovery Studio. Co-crystallized ligands were retained and prepared for re-docking as positive controls. Covalently bound ions within the protein structure were retained, and atoms were selected based on the highest occupancy. Missing polar hydrogens were added according to the protonation state at pH 7.0, and the atomic charges of all ions were manually adjusted based on their valency. The cleaned protein structures were saved in PDB format and subsequently converted into AutoDock-compatible PDBQT files using AutoDock Tools (ADT). Docking simulations were performed in AutoDock4 (version 4.2.6) employing the Lamarckian Genetic Algorithm with 50 GA runs and a population size of 200. Grid maps were generated using AutoGrid 4.2 to define binding site parameters. Binding site selection was guided by the location of co-crystallized ligands or positive controls positioned within the active pocket of the target proteins. To validate the docking protocol, co-crystallized ligands from the three-dimensional structures of the target proteins were prepared and re-docked using the same computational procedures applied to the test compounds. Binding affinities and docking conformations were analyzed, and molecular interactions were visualized using BIOVIA Discovery Studio. Final docking results were evaluated based on binding energies, interacting residues, and chemical bonding patterns, providing a comprehensive assessment of the binding potential of Triphala-derived bioactive compounds against disease-associated targets [45]. This workflow ensured methodological rigor and reproducibility in evaluating compound–target interactions. AutoDock employs a semi-empirical free-energy force field to estimate binding thermodynamics according to the equation:

Δ*G* = VdW + H-bond + Electrostatics + Desolvation + Torsional Entropy

When Van der Waals (VdW): Evaluates dispersion and repulsion using a 6–12 Lennard-Jones potential, Hydrogen Bonding (H-bond): Measures directional atom–atom interactions (10–12 potential), Electrostatics: Calculates charge–charge interactions via a distance-dependent Coulombic potential., Desolvation: Estimates the energetic penalty of removing water shells from polar/apolar atoms. Torsional Entropy: Accounts for the loss of conformational flexibility upon ligand binding.

### 2.14. Molecular Dynamics Simulation

Molecular dynamics simulations were performed to investigate the time-dependent behavior of Kynurenic acid-ACE and Kynurenicacid-PTGS2 complexes, providing insights into ligand-induced effects on protein flexibility and binding. Complexes were prepared at pH 7.0 using the Protein Preparation Wizard (Schrödinger Release 2022-3: Maestro, Schrödinger, LLC, New York, NY, USA), which added hydrogens, assigned bond orders, rebuilt missing side chains and loops, optimized hydrogen-bond networks, and sampled water orientations. The OPLS4 force field was applied, and each system was solvated in an orthorhombic TIP3P water box (10 Å × 10 Å × 10 Å), neutralized with 0.15 M Na+ and Cl− ions, and parameterized for simulation. Production runs of 200 ns were conducted under an NPT ensemble at 310 K and 1.01 bar, with long-range electrostatics treated using the Smooth Particle Mesh Ewald (PME) method and solvent modeled by a simple point-charge representation. Trajectory analyses—including RMSD profiles, RMSF plots, ligand–protein contact maps, and timeline interaction analyses—were performed using the Simulation Interaction Diagram Wizard. All simulations and analyses were carried out in Desmond (Schrödinger Release 2022-3: Maestro), providing a comprehensive view of structural stability, conformational dynamics, and key interaction hotspots throughout the simulation [46].

### 2.15. Statistical Data Analysis

All experiments were performed in triplicate, and data were expressed as the mean ± standard deviation (SD). Statistical analysis was carried out using GraphPad Prism (Version 10) (GraphPad Prism Software, San Diego, CA, USA). An independent *t*-test was used to determine significant differences between groups. A *p*-value of less than 0.05 was considered statistically significant.

## 3. Results

### 3.1. LC-MS and GC-MS Phytochemical Profile

Phytochemical profiling of the boiled aqueous Triphala extract was performed using complementary LC-MS and GC–MS analyses to characterize both polar and volatile metabolites. Compound identification was based on a combination of chromatographic retention time (RT), accurate molecular mass, characteristic ions (*m*/*z*), molecular formula, and spectral matching against reference mass spectral libraries.

The key marker antioxidant compounds of Triphala extract were determined by using LC-MS and GC-MS (Table 1 and Table S1). LC-MS demonstrated that the phenolic compound, gallic acid was abundant along with pyrogallol and trans-cinnamic acid. 4-Methoxychalcone, the flavonoid was identified. Triphala extract was rich in terpenoid compounds, especially cyclofenchene and Carene (Table S1). Amino acid and their derivatives constitute in Triphala extract include arginine and aspartic acid. For GC-MS results presented the boiled-stable volatile and semi-volatile contents. The phenolic component pyrogallol was detected as the dominant compound, with 56.24% relative abundance, together with 3-Dihydro-3,5-dihydroxy-6-methyl-4H-pyran-4-one, trans-Cinnamic acid, and 2,4-Di-tert-butylphenol. The results of LC-MS and GC-MS demonstrate that Triphala extract contains the bioactive metabolites. The key bioactive compounds were gallic acid and pyrogallol, which reflect polyphenol content and antioxidant capacity. Together, these markers are associated with antioxidant, anti-inflammatory, antimicrobial, and lipid-regulatory activities.

### 3.2. Antioxidant Radical Scavenging Activity

**Figure 1:**
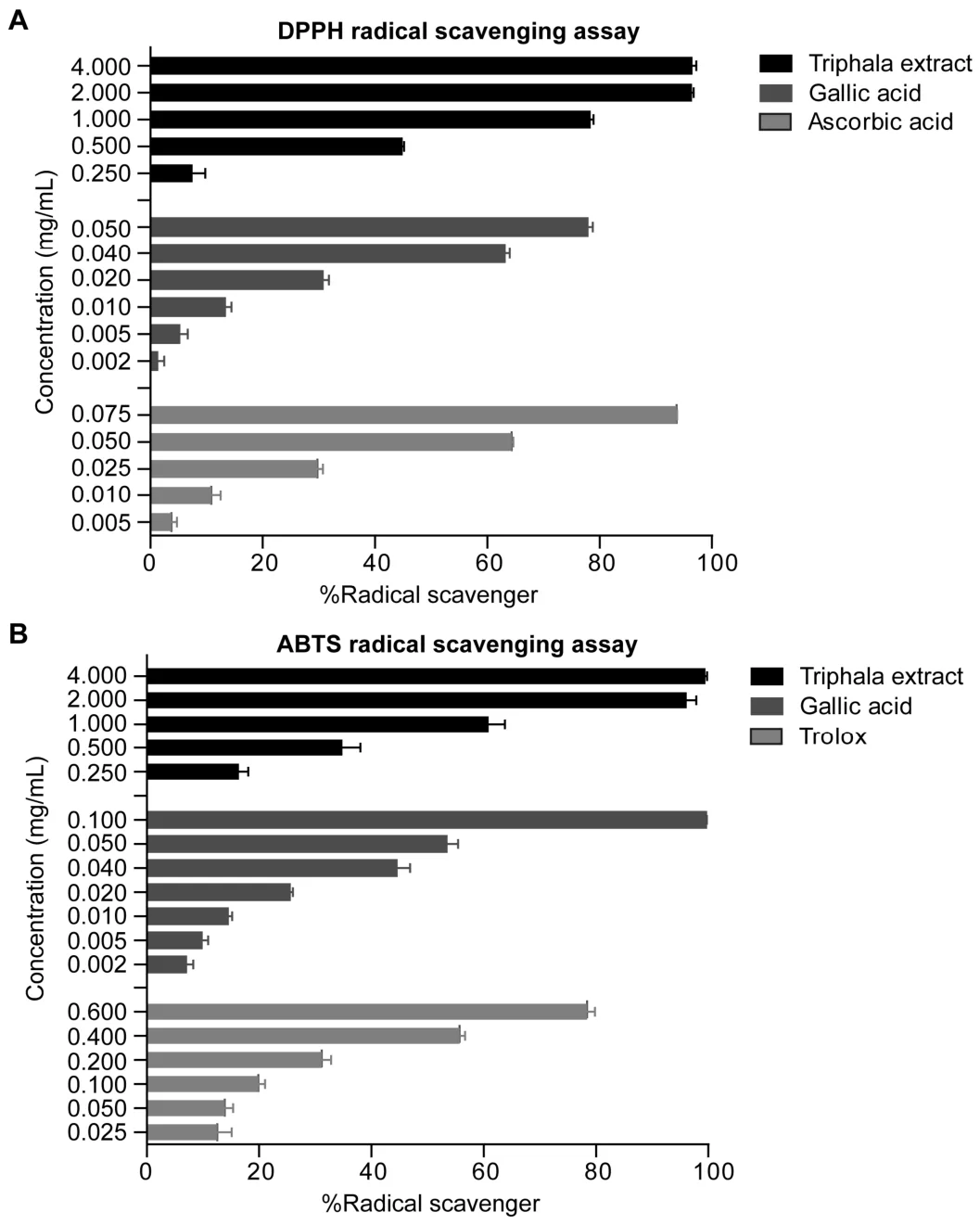
Antioxidant radical scavenging activity of Triphala extract (**A**) DPPH with an IC_50_ of approximately 0.6 mg/mL, ascorbic acid and gallic acid have been used as positive controls. (**B**) ABTS assay with an IC_50_ of approximately 0.8 mg/mL of the TP extract, Trolox, and gallic acid have been used as positive controls.

The intrinsic antioxidant potential of the Triphala aqueous extract was evaluated by the ability to scavenge DPPH and ABTS radicals. The TP extract exhibited considerable DPPH radical scavenging activity with IC_50_ value of about 0.6 mg/mL as shown in Figure 1. The pure positive control, ascorbic acid and gallic acid, had IC_50_ values below 0.1 mg/mL. ABTS radical scavenging assay showed the IC_50_ of TP extract 0.8 mg/mL, indicating strong antioxidant capacity. However, gallic acid remained the most potent antioxidant with an IC_50_ value of about 0.05 mg/mL, while Trolox showed an IC_50_ of approximately 0.35 mg/mL.

### 3.3. Hemolytic Activity of Extracts on Normal and G6PD-Deficient Human RBC

**Figure 2:**
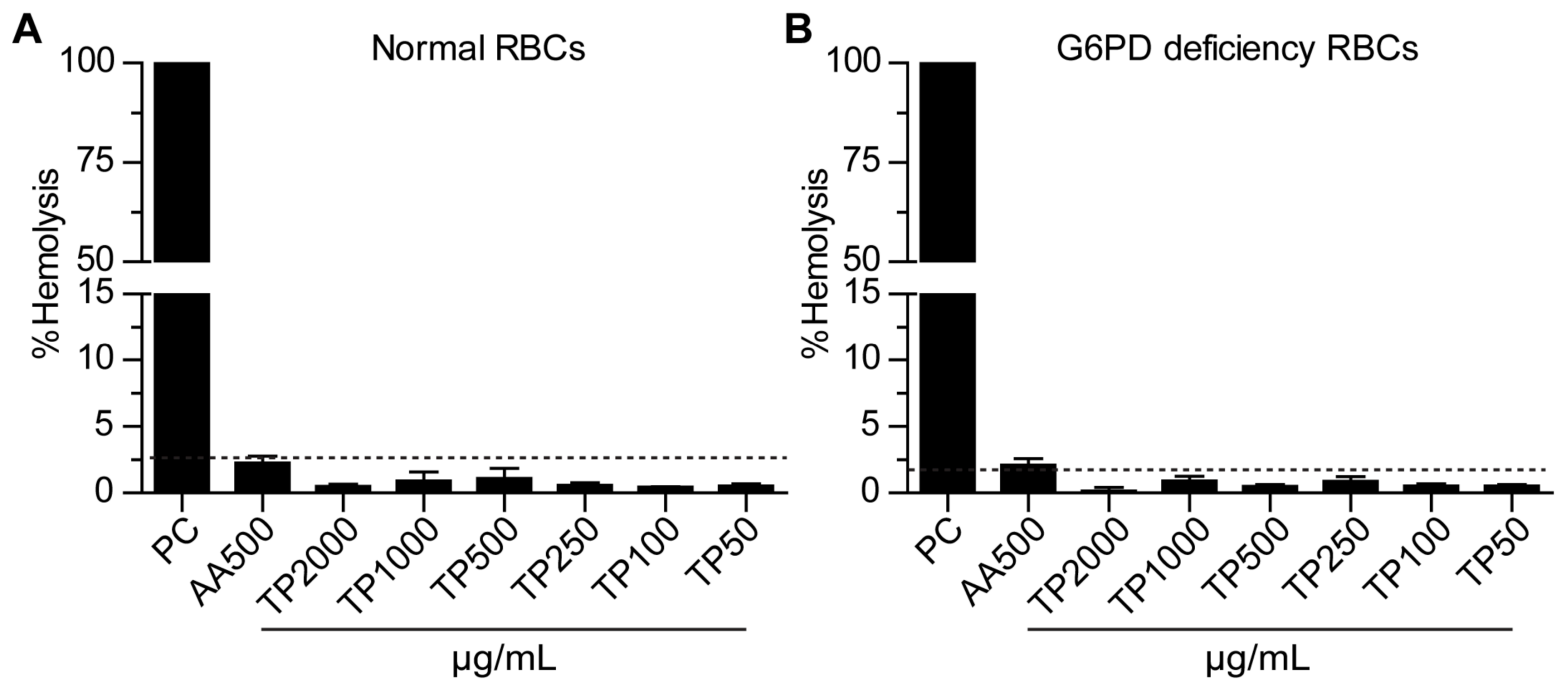
Hemolyticactivity of extracts on RBCs. The TP extract concentrations of 25–2000 µg/mL showed no significant effect on %hemolysis under both conditions (**A**) Normal RBCs and (**B**) G6PD-deficient RBCs. The values showed as mean ± SD. The statistical significance was tested compared to the Distilled water used as a positive control. The dotted line represented the baseline percent hemolysis in PBS (negative control) at 2.63 (normal RBCs) and 1.98 (G6PD-deficiency RBCs), respectively. Dashed line represents the baseline hemolysis (%) in PBS.

To investigate the safety of Triphala extract on RBC, normal and G6PD-deficient RBCs were used. Triphala extract in various concentrations was incubated with RBC. The DW was used for hemolysis control (100% hemolysis), and the pure ascorbic acid (AA) was used as the test control. The result showed that both ascorbic acid and triphala extract did not affect normal and G6PD deficient-RBC, with %hemolysis lower than 5% in all concentrations (Figure 2). The dotted line represented the baseline percent hemolysis in PBS (negative control) at 2.63 (normal RBCs) (Figure 2A) and 1.98 (G6PD deficiency RBCs) (Figure 2B), respectively.

### 3.4. Protective Effect of Triphala on H_2_O_2_-Induced Hemolysis

**Figure 3:**
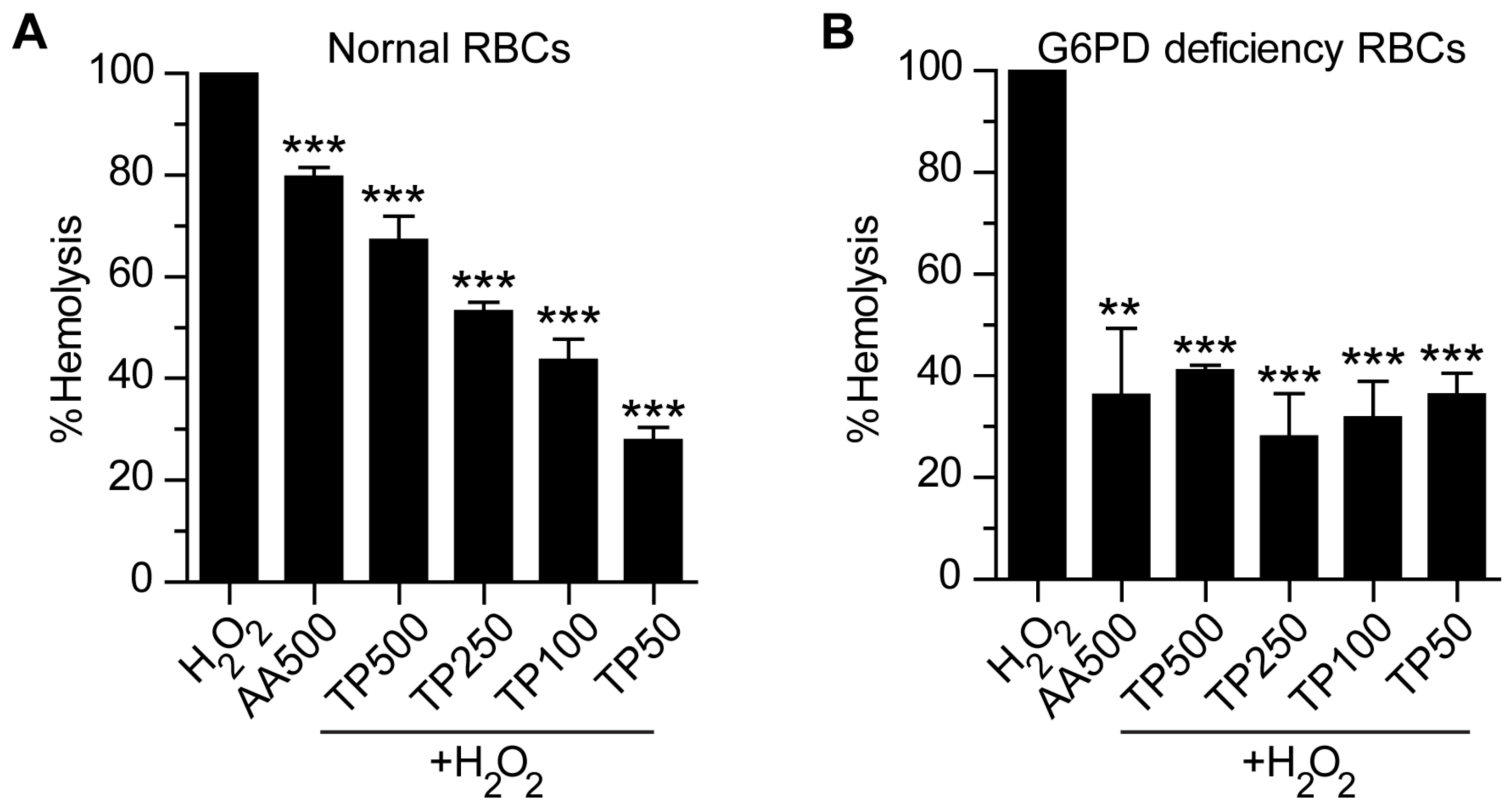
Effect of Triphala on H_2_O_2-_induced hemolysis. (**A**) Normal RBCs; The protective effect on the oxidative stress against H_2_O_2_-induced hemolysis corresponds to the dose-dependent inhibition, ranging from 50–500 µg/mL. (**B**) G6PD-deficient RBCs; All concentrations of the Triphala extract, ranging from 25–500 µg/mL, provided statistically significant protection against H_2_O_2_ compared to positive control. The value shown as Mean ± SD. The statistics significant were analyzed by comparing to H_2_O_2_-treated condition (**, *p* < 0.01; ***, *p* < 0.001).

To investigate the RBC protective effect of Triphala extract in oxidative stress-induced conditions. For normal red blood cells, the Triphala extract showed the dose-dependent protection against H_2_O_2_-induced oxidative damage (Figure 3). Normal RBC exposure to H_2_O_2_, the TP extract significantly reduced the hemolysis. The most effective protection was shown at a concentration of 50 µg/mL, with hemolysis reduced to 34.64% (*p* < 0.05) compared to the positive control condition (Figure 3A). Interestingly, the efficacy followed a bell-shaped curve in this model, as higher concentrations (such as 500 µg/mL) showed a relative increase in hemolysis to 67.21%, suggesting that optimal membrane stabilization in healthy cells occurs at lower, specific dosages.

For the protective effect on the oxidative stress defense defect model, G6PD-deficient RBCs were used. The protective capacity of Triphala was significantly efficient in the G6PD-deficient RBC (Figure 3B). All concentrations of the Triphala extract, ranging from 25 to 500 ug/mL, provided statistically significant protection against H_2_O_2_ compared to the positive control (*p* < 0.05). The maximum protective effect was 250 µg/mL, where hemolysis was only 27.94%. At the lowest concentration of 25 µg/mL, the hemolysis was 45.50% (Figure 3 and Supplement Table S2).

### 3.5. Drug-likeness Prediction of Compound-Derived from Triphala Extracts

The drug-likeness evaluation of Triphala-derived compounds highlights how their structural classes influence pharmacological potential. As shown in Table S3, the phenolic compounds, pyrogallol and gallic acid, both satisfied Lipinski’s criteria but triggered one PAINS alert due to their polyhydroxylated aromatic structures, which are prone to assay interference. In contrast, the amino acid derivatives, including pyroglutamic acid, N-acetylglutamic acid, pipecolic acid, and kynurenic acid, showed favorable physicochemical properties, with molecular weights under 200 g/mol, balanced hydrogen bond donors/acceptors, and no PAINS alerts, suggesting good drug-like potential. The aromatic carboxylic acid, trans-cinnamic acid, also complied with all drug-likeness filters, reinforcing its suitability as a bioactive scaffold. The furan derivatives, including 5-hydroxymethylfurfural, furfural, and 2-furancarboxylic acid, demonstrated strong compliance with Lipinski, Veber, and Egan rules, with low molecular weights and favorable logP values, making them promising small heterocyclic candidates. The fatty acids, palmitic and stearic acid, met Lipinski’s criteria but showed high lipophilicity (logP > 5), leading to partial or failed compliance with Ghose, Veber, and Egan filters, which limits their drug-like potential despite their biological relevance. Finally, the lactone/cyclic compound, 2H-pyran-2,6-dione, exhibited excellent drug-likeness, passing all major filters with no PAINS alerts, reflecting its suitability as a reactive yet stable scaffold. Overall, the Triphala extract constituents range from highly drug-like small phenolics and heterocycles to less favorable long-chain fatty acids, underscoring their diverse physicochemical profiles and highlighting which groups are most promising for further pharmacological development. Overall, the Triphala extract constituents demonstrated promising drug-like properties, with several small phenolic and heterocyclic acids passing multiple drug-likeness filters, supporting their potential as bioactive candidates for further pharmacological investigation.

### 3.6. Pharmacokinetic (ADME) Prediction of Triphala-Derived Compounds

The ADME predictions revealed distinct pharmacokinetic behaviors across the chemical groups of Triphala compounds. As shown in Supplement Table S4, phenolic compounds such as pyrogallol and gallic acid showed moderate solubility and intestinal absorption (50–65%), but limited distribution and poor BBB penetration, indicating restricted CNS activity. In contrast, the amino acid derivatives (pyroglutamic acid, N-acetylglutamic acid, pipecolic acid, and kynurenic acid) demonstrated favorable absorption (25–95%), balanced hydrogen bonding, and moderate systemic distribution. Kynurenic acid was notable for its CYP1A2 inhibition, suggesting potential drug–drug interaction risks, while the others showed minimal metabolic liabilities. The aromatic carboxylic acid, trans-cinnamic acid, exhibited excellent intestinal absorption (~97%) and moderate clearance, reinforcing its drug-like potential. The furan derivatives (5-hydroxymethylfurfural, furfural, and 2-furancarboxylic acid) displayed excellent absorption (96–100%) and favorable permeability, with no CYP interactions predicted, supporting their suitability as safe drug-like scaffolds. Conversely, the fatty acids (palmitic and stearic acid) were limited by very poor solubility but retained high intestinal absorption (>90%). Their high lipophilicity contributed to broader tissue distribution and higher clearance values, though CYP1A2 inhibition raised concerns about metabolic interactions. Finally, the lactone compound 2H-pyran-2,6-dione demonstrated strong drug-like properties, with complete intestinal absorption, favorable solubility, and no CYP-related liabilities (Supplement Table S4). Overall, Triphala constituents showed consistently high absorption and low metabolic risks, with phenolics, amino acid derivatives, and furan compounds emerging as promising drug-like candidates, while fatty acids were less favorable due to solubility and lipophilicity constraints. This highlights the chemical diversity of Triphala extracts and identifies specific groups with the most potential for pharmacological development.

### 3.7. Toxicity Prediction of Triphala-Derived Compounds

The toxicity predictions of Triphala-derived compounds revealed generally favorable safety profiles, with only a few compounds showing potential risks. As shown in Supplement Table S5, phenolic compounds (pyrogallol and gallic acid) were non-mutagenic in the AMES test, exhibited moderate tolerated doses, and showed no hepatotoxicity or skin sensitization. Similarly, amino acid derivatives (pyroglutamic acid, pipecolic acid, and kynurenic acid) were predicted to be safe, with high tolerated doses and no major toxicity alerts. However, N-acetylglutamic acid was flagged as AMES positive, indicating mutagenic potential, which may limit its suitability for therapeutic use. The aromatic carboxylic acid trans-cinnamic acid and furan derivatives (5-hydroxymethylfurfural, furfural, and 2-furancarboxylic acid) showed excellent tolerated doses and low toxicity overall, though cinnamic acid and furfural were predicted to cause skin sensitization. The fatty acids (palmitic and stearic acid) demonstrated poor water solubility but high tolerated doses; both were associated with skin sensitization, and stearic acid showed weak hERG II inhibition, suggesting possible cardiotoxicity risks. Finally, the lactone compound 2H-pyran-2,6-dione was predicted to be safe in most parameters but showed potential for skin sensitization (Supplement Table S5). Overall, Triphala compounds exhibited low mutagenicity, hepatotoxicity, and cardiotoxicity risks, with phenolics, amino acid derivatives, and furan compounds emerging as the safest groups. Fatty acids and certain sensitizing compounds require caution, but the majority of Triphala constituents demonstrated promising safety profiles for further pharmacological exploration.

### 3.8. Prediction of Common Targets and Analysis

**Figure 4:**
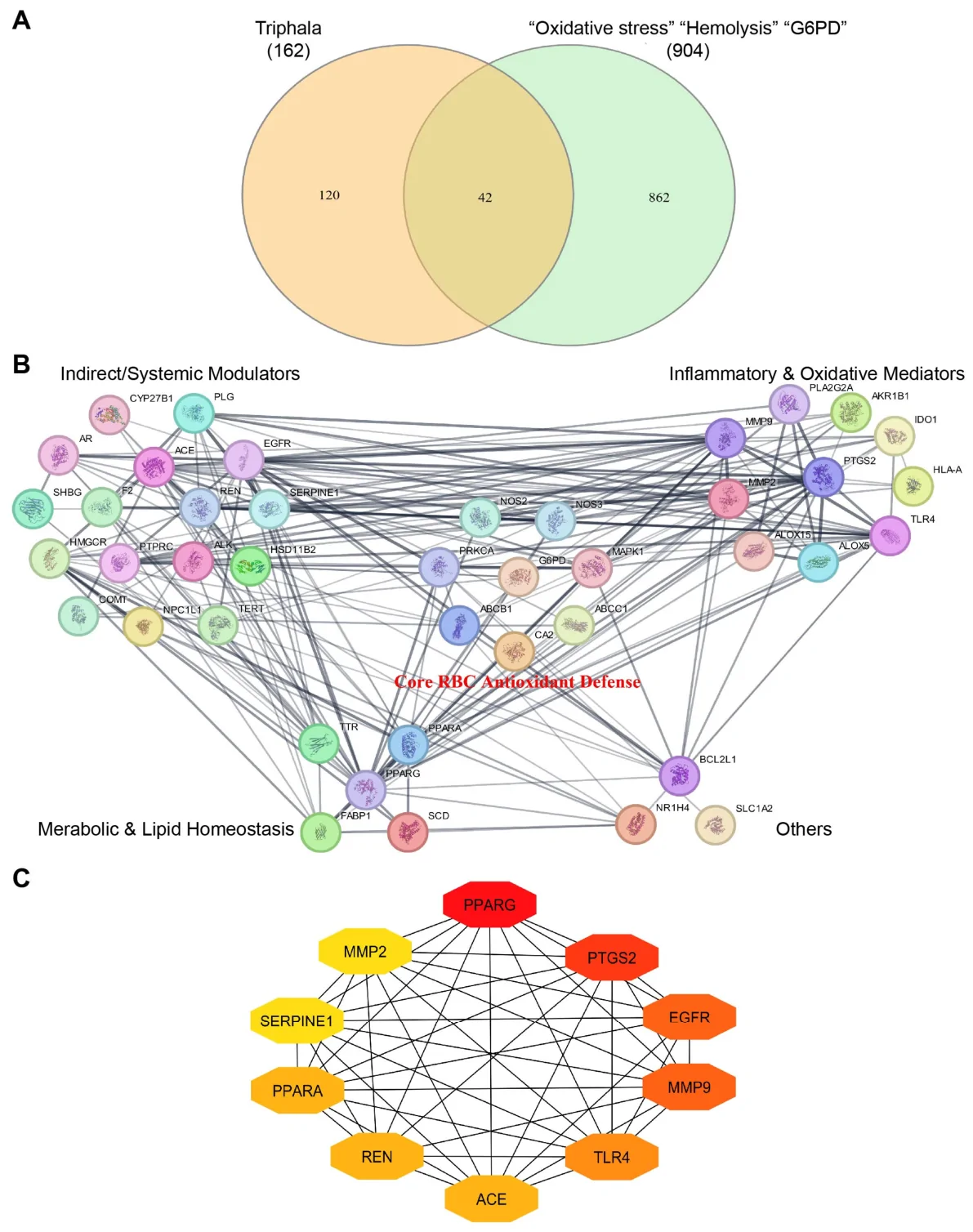
Overlapping targets of Triphala-derived compounds. PPI network of 42 common genes, and the top 10 hub genes. (**A**) Overlapping targets of Triphala-derived compounds involved in antioxidant defense against RBC hemolysis in normal and G6PD-deficient models. (**B**) Protein–protein interaction (PPI) network of 42 overlapping genes identified as common targets of Triphala-derived compounds in antioxidant defense against RBC hemolysis. (**C**) Top 10 hub genes from the PPI network, determined using the CytoHubba plugin in Cytoscape based on degree centrality (DC) values.

The integration of compound–target prediction with oxidative stress and hemolysis-related gene sets revealed a notable overlap between Triphala-derived compounds and RBC antioxidant defense pathways in both normal and G6PD-deficient models. From the Triphala dataset, 162 potential targets were identified, while 904 genes were associated with oxidative stress, hemolysis, and G6PD deficiency. As illustrated in Figure 4A, 120 targets were unique to Triphala and 862 were specific to oxidative stress/hemolysis/G6PD, whereas 42 targets were shared between the two datasets. These overlapping proteins represent key molecular mediators that might link the pharmacological actions of Triphala to RBC protection under oxidative stress. Importantly, this shared subset provides a focused pool of candidates for functional characterization and experimental validation, underscoring the potential of Triphala to exert multi-target antioxidant effects in hemolysis models.

### 3.9. Results of PPI Network Construction

To investigate the interactions among the 42 overlapping targets of Triphala-derived compounds involved in antioxidant defense against RBC hemolysis in normal and G6PD-deficient models, a PPI network was constructed using the STRING database. As shown in Figure 4B, the network depicts the complex interconnections among these proteins, with each node representing a target and edges denoting experimentally validated or predicted associations derived from curated databases, experimental evidence, and co-expression data. The thickness of the connecting lines reflects the strength of the interactions. The resulting network revealed a highly interconnected system, emphasizing the functional relationships among core RBC antioxidant defense proteins, including G6PD, NOS2/3, PRKCA, MAPK1, ABCC1/ABCB1, and CA2, directly regulate redox balance, glutathione recycling, ROS production, and eryptosis (Figure 4B and Supplement Table S6). In addition, several indirect/systemic modulators, such as ACE, REN, EGFR, AR, VDR, HMGCR, influence vascular tone, metabolism, and immune signaling, while inflammatory and oxidative mediators (PTGS2, ALOX5/15, PLA2G2A, MMP2/9, TLR4, IDO1, AKR1B1) amplify oxidative damage through prostaglandin synthesis, lipid peroxidation, and immune activation. Finally, metabolic and lipid regulators, including PPARA, PPARG, FABP1, SCD, and TTR, indirectly affect susceptibility to oxidative stress via lipid metabolism and antioxidant gene expression (Supplement Table S6). Together, these overlapping targets highlight the multi-layered mechanisms through which Triphala might exert protective effects, integrating direct RBC antioxidant defenses with systemic, inflammatory, and metabolic pathways.

### 3.10. Top 10 Targets Identification by CytoHubba

To identify the most influential regulatory proteins within the Triphala–RBC antioxidant defense network, the CytoHubba plugin in Cytoscape 3.10.4 was applied to rank hub genes according to their degree of connectivity. This analysis highlighted ten highly interconnected proteins, with node color intensity reflecting centrality, deeper shades indicating stronger connectivity (Figure 4C). The ranking results identified PPARG, PTGS2, EGFR, MMP9, TLR4, ACE, REN, PPARA, SERPINE1, and MMP2 as the top hub proteins. The prominence of these targets suggests that Triphala-derived compounds exert their protective effects by modulating multiple central regulators of oxidative stress, inflammation, and metabolic signaling, thereby influencing RBC survival under hemolytic conditions. Collectively, these findings underscore the multi-target nature of Triphala’s activity, acting through interconnected pathways rather than a single molecular mechanism. However, these hub proteins represent computational predictions and should be interpreted cautiously, as they do not yet constitute direct therapeutic targets. Additional cellular and in vivo validation will be required to substantiate these predictions and confirm their mechanistic relevance.

### 3.11. Functional Insights from Gene Ontology Enrichment

**Figure 5:**
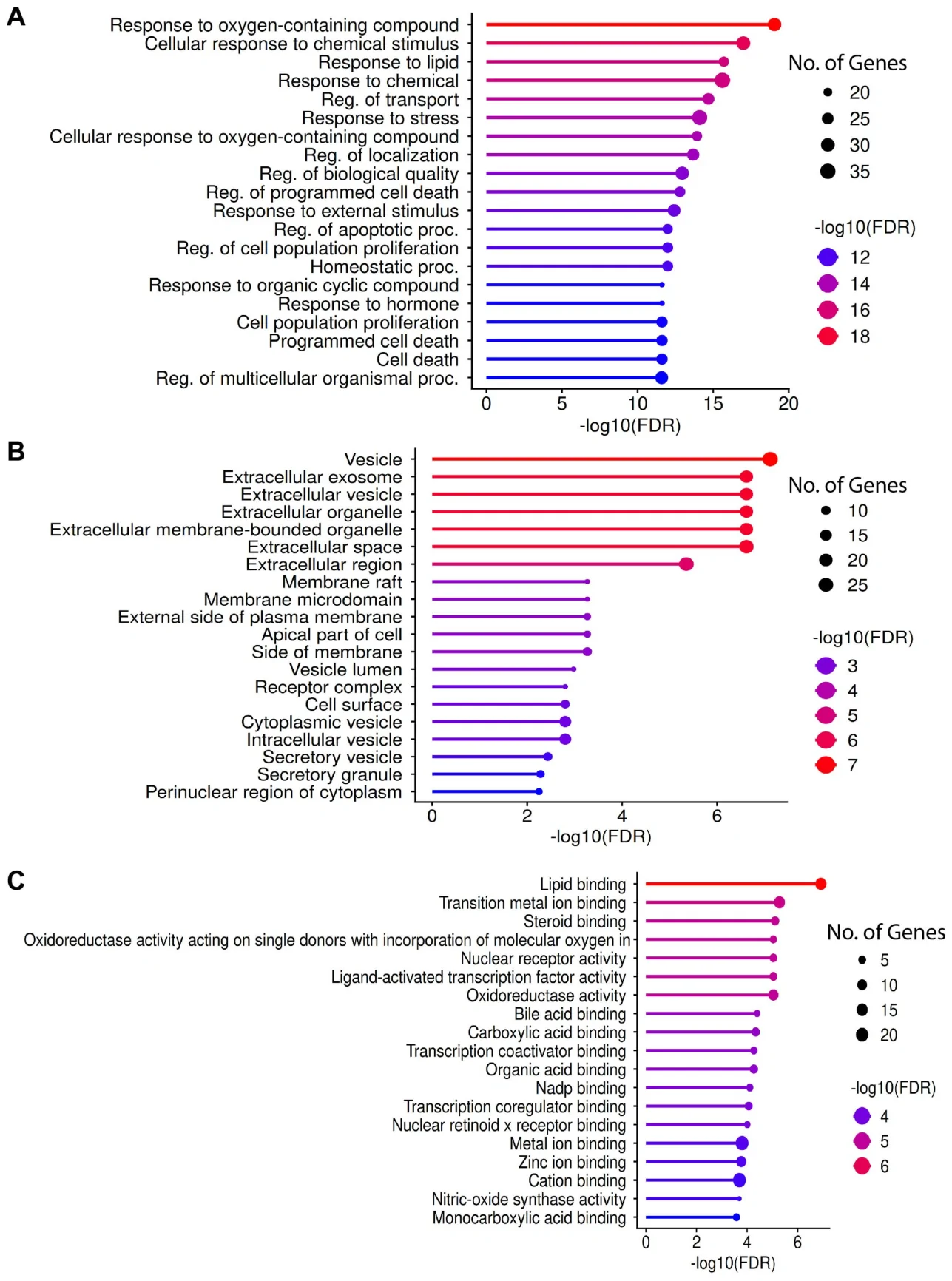
Gene Ontology (GO) enrichment analysis of Triphala-derived compounds associated with antioxidant defense against RBC hemolysis in normal and G6PD-deficient models (*p* < 0.05), showing enriched terms for Biological Process (**A**), Cellular Component (**B**), and Molecular Function (**C**).

The Gene Ontology enrichment analysis of the 42 shared targets of Triphala-derived compounds revealed strong associations with oxidative stress responses and cell survival pathways. In the Biological Process category (GOBP), highly significant terms included response to oxygen-containing compound (GO:1901700), regulation of biological quality (GO:0065008), response to lipid (GO:0033993), cellular response to oxygen-containing compound (GO:1901701), response to stress (GO:0006950), regulation of cell death (GO:0010941), underscoring the role of these targets in redox balance and hemolysis protection (Figure 5A and Supplement Table S7). The Cellular Component (GOCC) analysis revealed significant enrichment in vesicle-related structures, including vesicles (GO:0031982), extracellular organelles (GO:0043230), extracellular membrane-bounded organelles (GO:0065010), extracellular exosomes (GO:0070062), and extracellular vesicles (GO:1903561) (Figure 5B and Supplement Table S7). These findings suggest that many of the shared targets are involved in intercellular communication and membrane-associated signaling processes that contribute to RBC stability under oxidative stress. In the Molecular Function (GOMF) analysis, enriched terms such as lipid binding (GO:0008289), oxidoreductase activity (GO:0016491), nuclear receptor activity (GO:0004879), steroid binding (GO:0005496), and NADP binding (GO:0050661) highlight the involvement of metabolic regulators and redox enzymes in antioxidant defense, underscoring their roles in maintaining RBC stability under oxidative stress (Figure 5C and Supplement Table S7). Collectively, these results demonstrate that Triphala acts through a multi-target network spanning oxidative stress responses, inflammatory signaling, and metabolic regulation, thereby supporting RBC survival under hemolytic conditions in both normal and G6PD-deficient models.

### 3.12. Biological Pathways Highlighted by KEGG Enrichment

**Figure 6:**
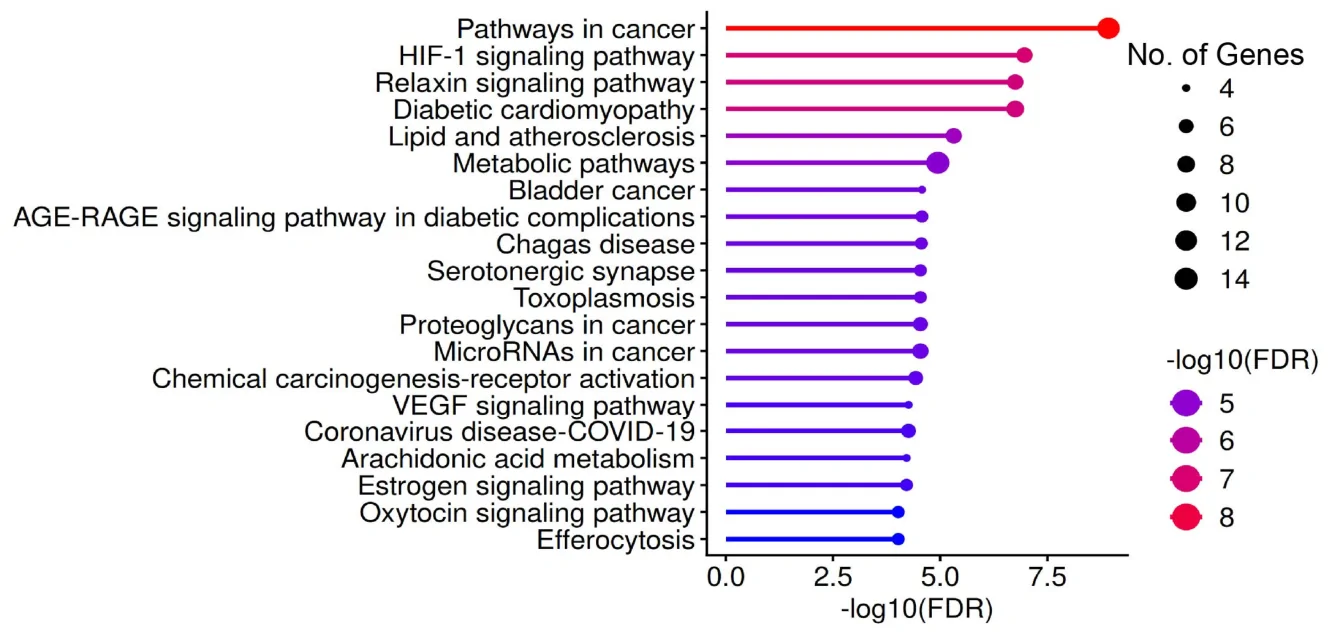
KEGG pathway enrichment analysis of genes associated with the antioxidant and anti-hemolytic effects of Triphala-derived compounds in normal and G6PD-deficient RBC models. Bars indicate fold enrichment, and colors represent −log10(FDR).

The KEGG pathway enrichment analysis of the 42 overlapping targets demonstrated that Triphala-derived compounds are associated with multiple signaling and disease-related pathways relevant to oxidative stress and RBC hemolysis. As shown in Figure 6 and Supplement Table S8, several pathways were directly linked to RBC function and vascular regulation. Among these, the HIF-1 signaling pathway (hsa04066) was enriched, reflecting its role in hypoxia response, erythropoiesis, and RBC survival, while the relaxin signaling pathway (hsa04926) highlighted vascular remodeling, endothelial function, and oxidative stress modulation. Cardiovascular-related pathways such as diabetic cardiomyopathy (hsa05415) and lipid and atherosclerosis (hsa05417) were also enriched, connecting oxidative damage, vascular dysfunction, and RBC fragility. In addition, the AGE-RAGE signaling pathway (hsa04933) underscored its contribution to vascular oxidative stress and RBC injury, and the VEGF signaling pathway (hsa04370) emerged as central to angiogenesis, vascular permeability, and oxygen delivery. Beyond the RBC and vascular-related pathways, significant enrichment was also observed in pathways in cancer (FDR = 3.22 × 10^−10^), along with other cancer-associated processes such as microRNAs in cancer, bladder cancer, and proteoglycans in cancer, as well as immune-related pathways. As shown in Supplement Table S8, these cancer-related pathways represented the most statistically significant enrichments. Although they may appear less directly connected to RBC hemolysis and oxidative stress, their inclusion reflects shared molecular mechanisms. Collectively, these findings suggest that Triphala might exert protective effects against RBC hemolysis through multi-pathway modulation, integrating hypoxia signaling, vascular regulation, oxidative stress control, and systemic metabolic responses.

### 3.13. Docking Evaluation of Hub Proteins

Molecular docking analysis was performed to evaluate the binding affinity of Triphala-derived compounds against the top 10 hub proteins implicated in antioxidant defense in RBC (Table 2). Docking analysis of the top ten hub proteins revealed distinct interaction patterns across functional groups. Within the core RBC antioxidant defense, nuclear receptors PPARG and PPARA showed moderate to strong binding with several Triphala compounds. In particular, kynurenic acid exhibited high affinity for PPARG (−6.5 kcal/mol) and PPARA (−5.62 kcal/mol), suggesting its role in enhancing lipid metabolism and antioxidant gene expression. As shown in Table 2, other compounds such as octadecanoic acid (stearic acid) and trans-cinnamic acid also demonstrated favorable affinities, reinforcing their contribution to stabilizing redox balance and protecting RBCs against oxidative stress.

For the inflammatory and oxidative mediators, including PTGS2 (COX-2), MMP9, MMP2, and TLR4, Triphala compounds consistently showed relevant binding. Kynurenic acid again stood out, with strong interactions at PTGS2 (−7.45 kcal/mol) and MMP9 (−6.42 kcal/mol), indicating potential inhibition of prostaglandin synthesis and matrix remodeling that exacerbate oxidative damage. Moderate affinities of trans-cinnamic acid and octadecanoic acid across these mediators further suggest broad anti-inflammatory effects. In the group of indirect/systemic modulators (ACE, REN, EGFR, SERPINE1), kynurenic acid displayed strong binding to ACE (−7.12 kcal/mol) and EGFR (−6.05 kcal/mol), implicating regulation of vascular tone and stress signaling (Table 2). Compounds such as pipecolic acid and gallic acid also showed moderate affinities, supporting systemic antioxidants and vascular protective mechanisms. Collectively, these results highlight that Triphala compounds act through multi-target binding across antioxidants, inflammatory, and systemic pathways, suggesting a coordinated protective mechanism against RBC hemolysis in both normal and G6PD-deficient models.

### 3.14. Binding Interactions of Kynurenic Acid and LPR702 (Co-Crystal Inhibitor) with ACE

**Figure 7:**
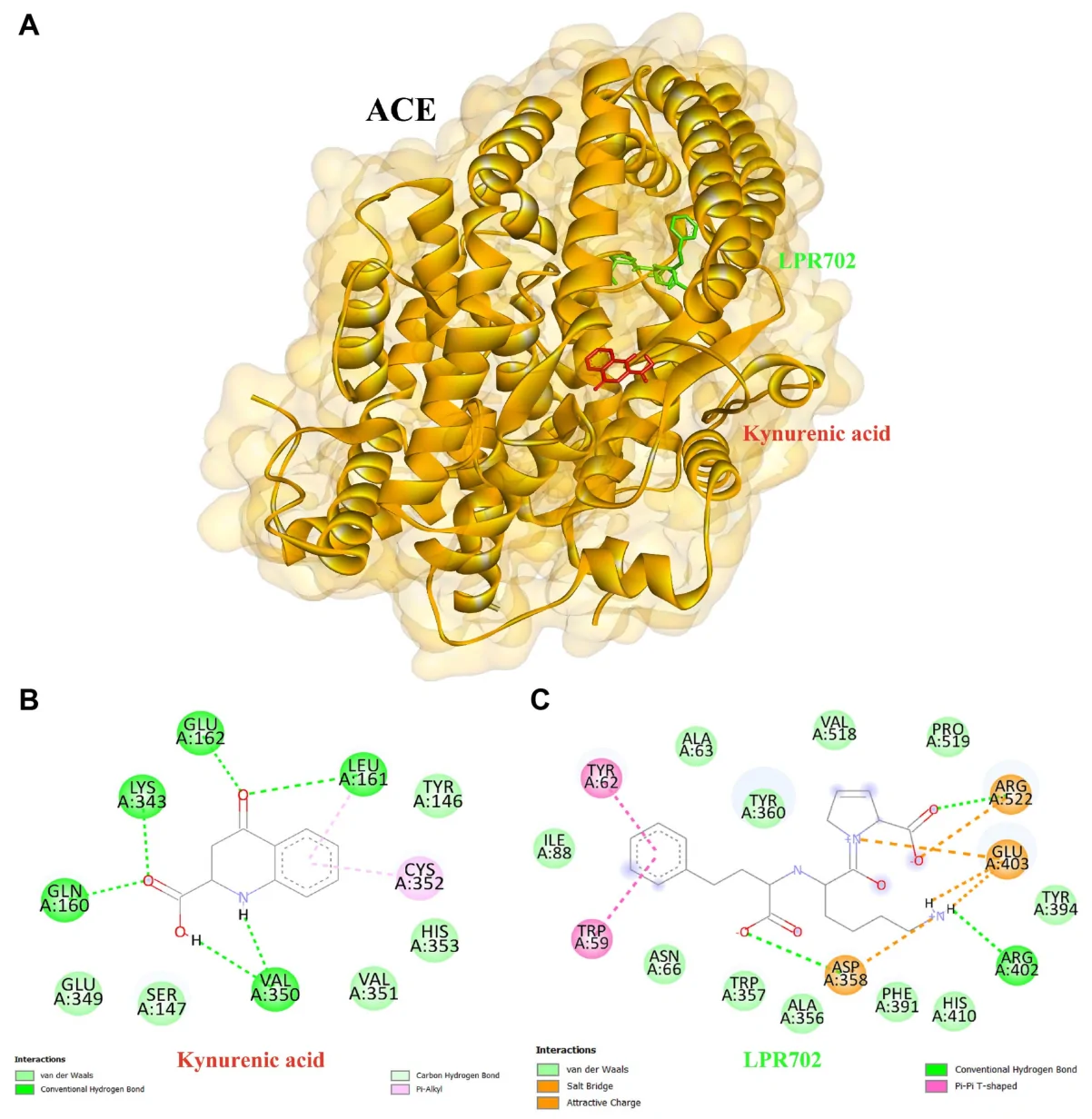
Visualization of ACE protein–ligand docking interactions. (**A**) Three-dimensional representation of ACE showing kynurenic acid (red) and LPR702 (co-crystal inhibitor, green) bound within the active site. (**B**) Two-dimensional interaction diagram of kynurenic acid with ACE, highlighting key residues and bonding types. (**C**) Two-dimensional interaction diagram of LPR702 with ACE, illustrating hydrogen bonds, electrostatic contacts, and aromatic interactions used as a positive control.

Molecular docking analysis revealed that kynurenic acid exhibited a binding affinity of less than −7.0 kcal/mol with ACE, which is stronger than that of the co-crystal inhibitor LPR702 (−5.73 kcal/mol) (Table 2). The binding interactions of kynurenic acid and LPR702 with ACE demonstrate distinct ligand–protein engagement within the enzyme’s active site (Figure 7A). Kynurenic acid formed van der Waals contacts with residues TYR146, SER147, GLU349, VAL351, and HIS353 (Figure 7B), along with conventional hydrogen bonds involving GLN160, GLU162, LEU161, LYS343, and VAL350. Additional stabilization was provided by Pi-alkyl interactions with LEU161 and CYS352. In contrast, LPR702 engaged a broader set of residues, including TYR62, TRP59, ASN66, ILE88, TYR360, TRP357, ASP358, GLU403, ARG522, HIS410, PHE391, and ARG394, through hydrogen bonds, salt bridges, attractive charge contacts, and Pi–Pi stacking (Figure 7C). Collectively, these binding profiles highlight that kynurenic acid achieves stable affinity primarily through hydrogen bonding and hydrophobic contacts, whereas LPR702 demonstrates stronger inhibitory potential via multiple electrostatic and aromatic interactions, underscoring complementary mechanisms of ACE inhibition.

**Figure 8:**
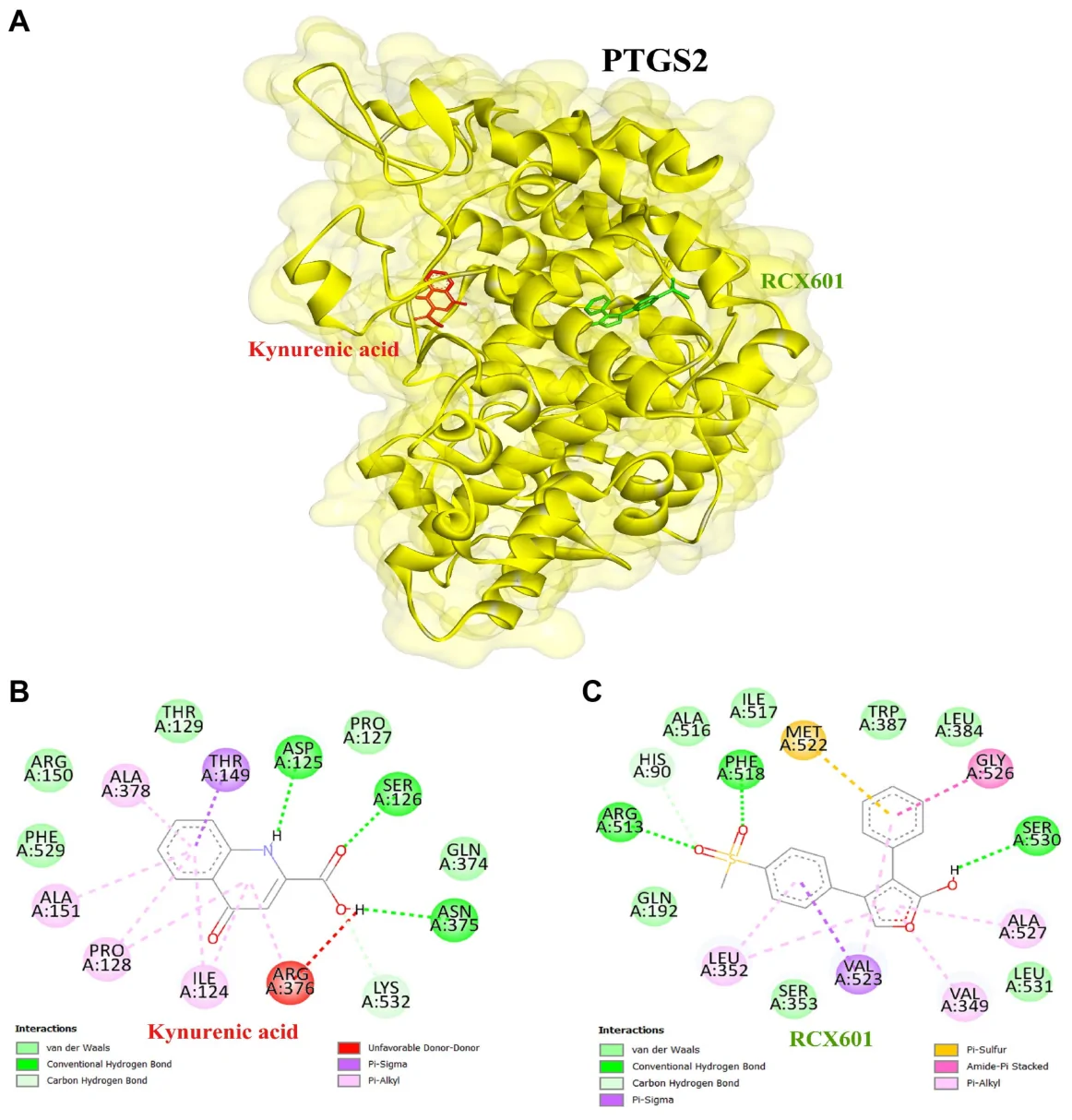
Docking interactions of Prostaglandin-Endoperoxide Synthase 2 (PTGS2) with ligands. (**A**) Three-dimensional representation of PTGS2 showing kynurenic acid (red) and RCX601 (co-crystal inhibitor, green) bound within distinct binding pockets. (**B**) Two-dimensional interaction diagrams of kynurenic acid with PTGS2, highlighting hydrogen bonds, van der Waals forces, and hydrophobic contacts. (**C**) Two-dimensional interaction diagrams of RCX601 with PTGS2, illustrating hydrogen bonding, electrostatic interactions, and aromatic contacts, serving as a positive control.

Molecular docking analysis revealed distinct ligand–protein engagements within the PTGS2 active site, with differences in binding sites, orientations, and interaction profiles (Figure 8A). Kynurenic acid interacts with PTGS2 through multiple stabilizing contacts, forming van der Waals interactions with PRO127, THR129, ARG150, GLN374, and PHE529 (Figure 8B). It also establishes conventional hydrogen bonds with ASP125, SER126, and ASN375, along with carbon hydrogen bonds involving LYS532. Hydrophobic stabilization is further supported by Pi-Sigma interactions with THR149 and Pi-Alkyl interactions involving ILE124, PRO128, ALA151, and ALA378, collectively providing a stable binding network within the PTGS2 active site. In contrast, RCX601 engages a broader set of residues with diverse interaction types (Figure 8C). It forms hydrogen bonds with HIS90, ARG513, PHE518, and SER530, van der Waals contacts with GLN192, SER353, LEU384, TRP387, ALA516, ILE517, and LEU531, and additional stabilizing interactions including Pi-Sigma (VAL523), Pi-Alkyl (ALA527, VAL349, LEU352), Pi-Sulfur (MET522), and amide-Pi stacking (GLY526). Collectively, these binding profiles highlight that kynurenic acid achieves stable affinity primarily through hydrogen bonding and hydrophobic contacts, underscoring complementary mechanisms of PTGS2 inhibition.

### 3.15. Binding Ability of Kynurenic Acid with ACE and PTGS2 Target Protein Using MD Simulation

**Figure 9:**
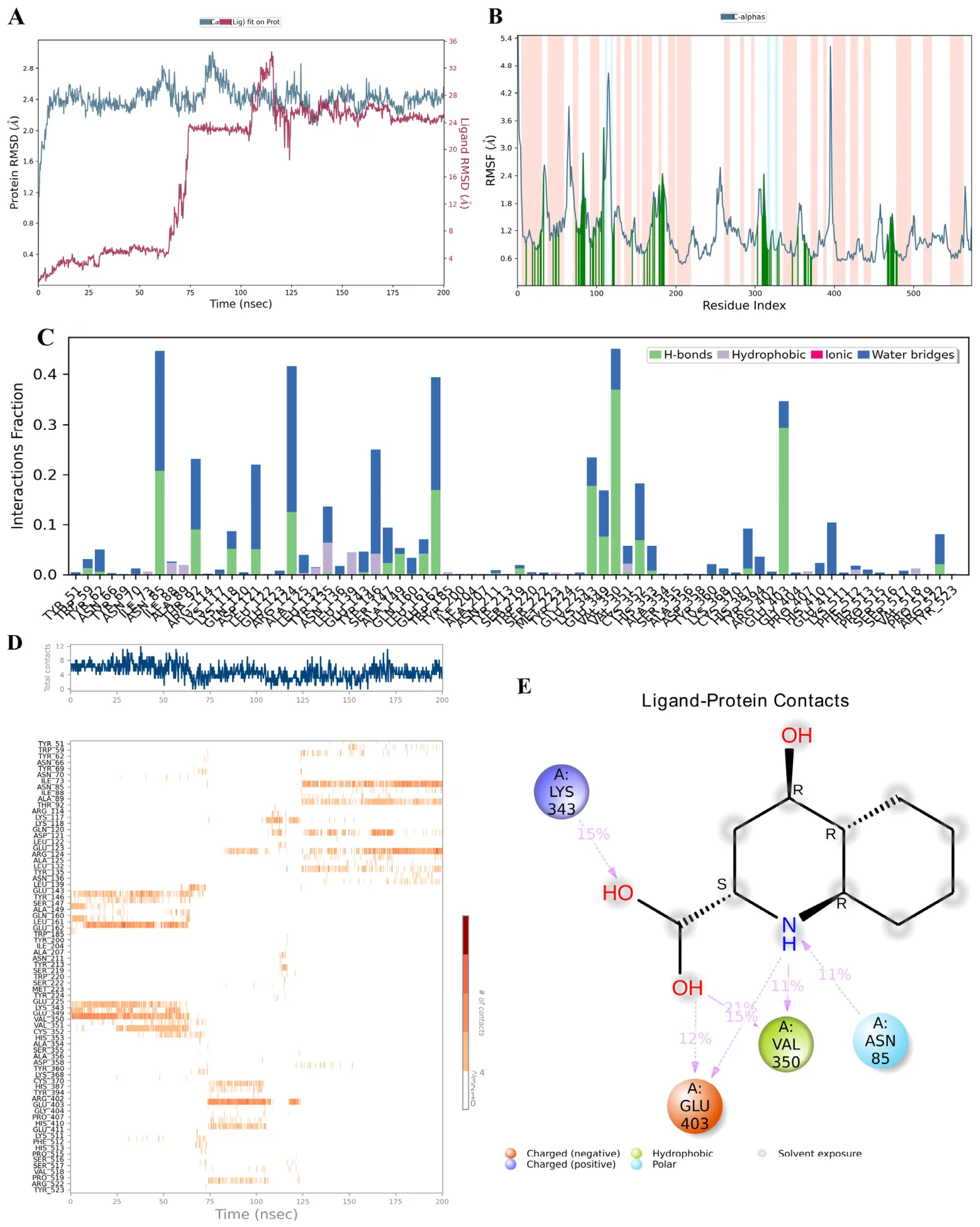
Binding capability of kynurenic acid with ACE protein analyzed by molecular dynamics simulation, showing RMSD (**A**), RMSF values and protein residues interacting with the ligand are indicated by green vertical bars. α‑Helical regions and β‑strand regions are highlighted with red and blue backgrounds, respectively (**B**), protein–ligand contact plots (**C**), MD timeline interaction analysis (**D**), and 2D interaction diagram of kynurenic acid with ACE protein (**E**).

To validate the molecular docking results, MD simulations were performed to assess the binding stability of kynurenic acid, a Triphala-derived compound, with the ACE and PTGS2 hub proteins under conditions mimicking the physiological environment of human cells. The ACE backbone remained stable throughout the 200 ns simulation, with RMSD values plateauing near 2.4 Å, while kynurenic acid exhibited a distinct RMSD shift around 75 ns before stabilizing, indicating a conformational adjustment within the binding pocket (Figure 9A). The RMSF analysis of the ACE protein in complex with kynurenic acid revealed overall structural stability, with most residues showing fluctuations below 2 Å, indicating limited flexibility across the backbone. Notable peaks were observed in specific loop regions, reflecting localized flexibility, while the majority of residues remained stable, supporting the idea that kynurenic acid binding does not induce major conformational changes but allows adaptive movements within dynamic regions of ACE (Figure 9B). The interaction fraction analysis of kynurenic acid with ACE showed that the ligand maintained consistent contacts with several key residues throughout the simulation, with fractions indicating recurrent hydrogen bonding and hydrophobic interactions. Residues such as ASN85, THR92, ASP121, ARG124, GLU162, LYS343, VAL350, and GLU403 contributed prominently to the overall interaction profile, reflecting their importance in stabilizing the ligand within the binding pocket. The sustained fractions across these residues highlight the dynamic yet stable nature of kynurenic acid–ACE binding, supporting its potential modulatory role in ACE activity (Figure 9C). The protein–ligand contact timeline analysis of kynurenic acid with ACE revealed dynamic yet sustained interactions across the 200 ns simulation. The total number of contacts fluctuated during the early phase (0–75 ns) and mid-phase (75–125 ns), while the heatmap highlighted key residues such as ASN85, THR92, and ARG124 that established recurrent interactions with the ligand during the later phase (125–200 ns). These persistent contacts indicate that kynurenic acid remains stably accommodated within the ACE binding pocket, with specific residues contributing to long-term stabilization and recognition throughout the trajectory (Figure 9D). The 2D interaction analysis of kynurenic acid with ACE revealed stable contacts with several key residues, including LYS343, GLU403, VAL350, and ASN85, which contributed significantly to ligand stabilization within the binding pocket. The interaction fractions, ranging between 11–21%, indicate recurrent hydrogen bonding and electrostatic interactions that reinforce the binding affinity of kynurenic acid, highlighting its potential modulatory role in ACE activity (Figure 9E).

**Figure 10:**
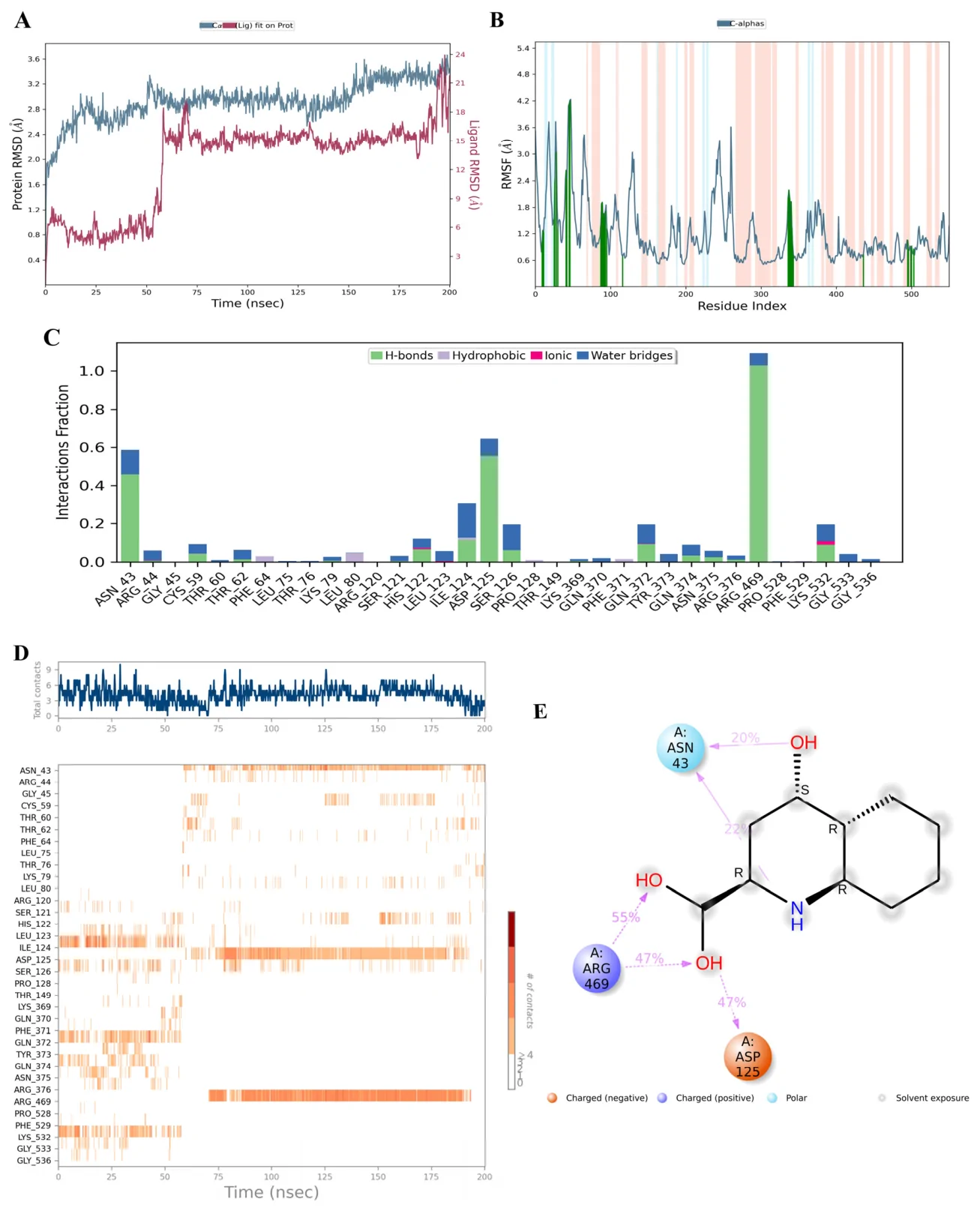
Binding capability of PTGS2–kynurenic acid complex analyzed by molecular dynamics simulation, showing RMSD (**A**), RMSF values and protein residues interacting with the ligand are indicated by green vertical bars. α‑Helical regions and β‑strand regions are highlighted with red and blue backgrounds, respectively (**B**), protein–ligand contact plots (**C**), MD timeline interaction analysis (**D**), and 2D interaction diagram of kynurenic acid with the PTGS2 protein (**E**).

The RMSD analysis of the PTGS2–kynurenic acid complex showed that the protein backbone remained stable during the 50–200 ns simulation, with Cα RMSD values fluctuating moderately before stabilizing around 3.0 Å. In contrast, the ligand exhibited higher variability, with a noticeable increase in RMSD near 50 ns followed by stabilization, suggesting that kynurenic acid underwent conformational adjustments before achieving a stable binding mode within the PTGS2 pocket (Figure 10A). The RMSF analysis of the PTGS2–kynurenic acid complex showed that most residues exhibited fluctuations below 2.0 Å, indicating overall backbone stability during the 200 ns simulation. A few loop regions displayed higher peaks, reflecting localized flexibility, but the majority of residues remained stable, suggesting that kynurenic acid binding does not induce major conformational changes and instead allows adaptive movements within dynamic regions of PTGS2 (Figure 10B). The interaction fraction analysis of the PTGS2–kynurenic acid complex revealed stable and recurrent contacts with several key residues, including ASN43, ILE124, ASP125, SER126, GLN372, ARG469 and LYS532, which contributed prominently to ligand stabilization within the binding pocket. These residues maintained notable fractions of hydrogen bonding and hydrophobic interactions, underscoring their importance in supporting the dynamic yet stable nature of kynurenic acid binding (Figure 10C). Overall, the sustained interaction fractions highlight the adaptability of kynurenic acid within PTGS2 and its potential role in modulating the protein’s activity. The protein–ligand contact analysis of the PTGS2–kynurenic acid complex revealed that several residues maintained strong and recurrent interactions throughout the simulation. Notably, ASP125, and ARG469 showed higher interaction fractions, reflecting their key role in stabilizing the ligand within the PTGS2 binding pocket (Figure 10D). The 2D average protein–ligand contact analysis of the PTGS2–kynurenic acid complex revealed stable interactions with several key residues, most prominently ASN43, ASP125, and ARG469. These residues maintained significant contact percentages (ranging from ~20% to over 50%), reflecting recurrent hydrogen bonding and electrostatic interactions that contribute to ligand stabilization within the PTGS2 binding pocket (Figure 10E). Overall, the sustained average contacts highlight the strong and consistent engagement of kynurenic acid with PTGS2, supporting its potential role in modulating protein activity.

## 4. Discussion

Triphala is a traditional Ayurvedic herbal formulation composed of equal proportions of the dried fruits of *T*. *chebula*, *T*. *bellerica*, and *P*. *emblica*. It has been widely used for centuries in human health and is recognized for its diverse pharmacological activities [47,48]. The analysis of major compounds in Triphala is influenced by the extraction solvent, with water (aqueous) extraction, as used in the present study, primarily yielding highly polar phenolic compounds, particularly gallic acid and ellagic acid, along with hydrolysable tannins. This approach closely reflects traditional preparation methods and remains highly relevant for biological and clinical applications. Previous studies have demonstrated that aqueous extracts retain strong antioxidant activity through direct ROS scavenging, metal chelation, and modulation of endogenous antioxidant systems, despite having a relatively narrower chemical profile compared with organic solvent extracts. In our current work, LC-MS analysis demonstrated a compound profile dominated by phenolic acids and tannin derivatives, which is consistent with the previous findings, confirming that water extraction is sufficient to recover the principal antioxidant-active constituents of Triphala. In comparison, ethanol or hydroalcoholic extraction has been reported to enhance the extraction efficiency and chemical diversity of Triphala, particularly increasing the yield of hydrolysable tannins and flavonoids, such as chebulinic acid, chebulagic acid, and also quercetin derivatives, which are more soluble in organic solvents [47,48]. These compounds contribute to broader bioactivity and may strengthen antioxidant capacity in certain assay systems. However, comparative metabolomic analyses indicate that the core bioactive compounds remain largely consistent across extraction methods, with key constituents, including gallic acid, ellagic acid, chebulinic acid, and chebulagic acid, being detected in both water and ethanol-based extracts, albeit with differences in relative abundance [47]. Therefore, although ethanol extraction may provide a more comprehensive phytochemical profile, the findings from this study support that aqueous extraction captures the major functional compounds responsible for Triphala’s antioxidant effects. Collectively, these results suggest that the biological activity of Triphala is driven by a conserved core group of phenolic and tannin compounds, and that extraction method influences quantitative yield rather than fundamentally altering the qualitative phytochemical composition. Howerver, Triphala is a multi-component herbal formulation, batch-to-batch variability represents an important consideration for both research reproducibility and future clinical translation. Factors such as geographical origin, cultivation conditions, harvesting time, post-harvest processing, storage, and extraction methods may influence the phytochemical composition and consequently the biological activity of the extract. Therefore, rigorous standardization using chromatographic fingerprinting (e.g., LC-MS and GC-MS) and quantitative analysis of key bioactive compounds, such as gallic acid, ellagic acid, chebulagic acid, and chebulinic acid, is essential to ensure consistency across production batches. In addition, implementing standardized quality control protocols in accordance with Good Manufacturing Practice (GMP) principles would enhance the reproducibility of experimental findings and facilitate regulatory approval and clinical translation. Such an approach is critical for advancing Triphala from preclinical research toward evidence-based therapeutic applications through a “bench-to-bedside” translational framework.

Our work demonstrates that Triphala exhibits strong antioxidant activity, as evidenced by consistent findings across both DPPH and ABTS assays, indicating its effectiveness in scavenging free radicals through multiple mechanisms. From a biomedical perspective, the use of these two complementary assays is particularly valuable, as DPPH primarily reflects radical scavenging in an organic system, while ABTS is applicable in both aqueous and lipophilic environments. The agreement between these assays in the current study therefore suggests that Triphala contains functionally diverse antioxidant compounds capable of acting under different physicochemical conditions, which enhances its potential biological relevance. These findings are in line with previous studies reporting that Triphala possesses high antioxidant capacity across various in vitro models, largely attributed to its rich content of polyphenolic compounds, including phenolic acids and tannins [19,49]. Those compounds are widely recognized for their ability to donate electrons or hydrogen atoms, thereby neutralizing free radicals and preventing oxidative chain reactions. Importantly, several studies have shown that the antioxidant activity of Triphala is not limited to a single assay system but is consistently observed across different analytical platforms, supporting the robustness of its antioxidant potential. When compared with other studies, the antioxidant activity observed in the present study is comparable to previously reported results, particularly for water extracts of Triphala. Although some studies suggest that ethanol or hydroalcoholic extraction may yield higher total phenolic content, the overall antioxidant capacity remains largely similar, as the core bioactive compounds are conserved across extraction methods [47]. This is an important consideration for translational research, as the water extract used in this study reflects traditional preparation and practical application, yet still retains strong antioxidant properties. In addition, the consistent antioxidant performance of Triphala across different studies suggests a potential synergistic effect among its constituent compounds, instead of relying on a single dominant molecule. This synergistic effect may contribute to its relatively high and stable antioxidant capacity, even when variations in extraction method or experimental conditions are present. Taken together, our findings, together with existing publications, reinforce that Triphala is a potent natural antioxidant, with reliable activity across multiple assay systems and extraction approaches. This provides a strong foundation for further investigation into its biological effects, particularly in relation to oxidative stress-related conditions.

Triphala extract and its bioactive compounds have attracted considerable interest for their potential protective effects on red blood cells (RBCs), in particular under conditions of oxidative stress. From a hematological perspective, RBCs are naturally prone to oxidative damage due to their continuous exposure to oxygen, high membrane lipid content, and limited capacity for cellular repair [2,3,4,50]. This susceptibility is further pronounced in glucose-6-phosphate dehydrogenase (G6PD) deficiency, where impaired NADPH production compromises the regeneration of glutathione (GSH), the primary intracellular antioxidant, thereby increasing the susceptibility of RBCs to oxidative hemolysis [5]. Before evaluating the anti-hemolytic effects under oxidative stress conditions, the cytotoxicity of Triphala extract was assessed in both normal and G6PD-deficient RBCs. The results demonstrated that the percentage of hemolysis remained relatively consistent across all tested concentrations, indicating that Triphala extract did not induce significant membrane damage or cytotoxicity in erythrocytes. Similar observations have been reported for other medicinal plant extracts, including *Kalanchoe daigremontiana* Raym.-Hamet and H. Perrier and *Justicia spicigera* Schltdl., which exhibited low hemolytic activity and good erythrocyte biocompatibility under in vitro conditions [36]. These findings suggest that phenolic-rich plant extracts may possess favorable safety profiles toward RBC membranes at appropriate concentrations. In this context, the antioxidant activity of Triphala is mainly due to its high polyphenol content, which included gallic acid, ellagic acid, and hydrolysable tannins (chebulinic and chebulagic acids), are of particular relevance in supporting RBC integrity under oxidative stress [49,51]. In this study, Triphala reduced H_2_O_2_-induced hemolysis in both normal and G6PD-deficient RBCs, suggesting a potential anti-hemolytic effect under oxidative conditions. In normal RBCs, this effect was strongest at moderate concentrations (50–100 µg/mL), while lower and higher concentrations showed weaker effects. A similar trend was seen in G6PD-deficient RBCs, but the overall protective effect was likely due to their reduced antioxidant capacity. Higher concentrations (e.g., 500 µg/mL) showed less protection, suggesting a non-linear, dose-dependent response typical of phytochemical mixtures. Taken together, these findings suggest that Triphala may help reduce oxidative hemolysis, particularly at certain concentrations. However, the variation across concentrations and between RBC types suggests that its effects depend on both dose and the cell’s redox status. Therefore, although the findings are promising they should be interpreted with caution, and further studies are warranted to validate their consistency and physiological relevance. Our findings are generally in line with previous studies demonstrating that Triphala and its constituents possess antioxidant and cytoprotective properties, including the ability to reduce oxidative damage and support cellular redox balance [19,49]. Polyphenolic compounds in Triphala are known to exert direct radical scavenging activity, thereby reducing reactive oxygen species (ROS) and limiting oxidative injury to RBC membranes and hemoglobin. In addition, Triphala has been reported to support endogenous antioxidant systems, including enzymes, for example superoxide dismutase (SOD), catalase (CAT), and glutathione-related enzymes, which play important roles in detoxifying oxidative species [49,52]. The relatively reduced effect observed in G6PD-deficient RBCs in this study further aligns with the critical role of the GSH-dependent pathway in RBC defense, as these cells have a diminished capacity to counteract oxidative stress due to impaired NADPH production [5]. Interestingly, the biphasic response, where the higher concentrations of Triphala showed reduced effects, has also been reported for polyphenols, which can act as either antioxidants or pro-oxidants depending on concentration and cellular context. This phenomenon may be particularly relevant in hematological conditions such as G6PD deficiency and other hemolytic disorders, where RBCs are already under oxidative stress and may be more sensitive to subtle changes in redox balance. Overall, Triphala acts through multiple antioxidant mechanisms, including direct ROS scavenging and support of intracellular redox systems. These findings provide initial evidence of its anti-hemolytic activity in both normal and G6PD-deficient RBCs, while also emphasizing the importance of dose optimization and biological context. Further studies using well-defined RBC models and clinically relevant conditions are necessary to better understand its efficacy and safety in hematological applications.

To provide a systems-level view of Triphala’s interaction with pathways linked to oxidative stress-induced hemolysis and G6PD deficiency, in silico evaluation identified phenolics, amino acid derivatives, and furan compounds as the most promising drug-like candidates, while long-chain fatty acids showed poor compliance due to high lipophilicity. In silico analysis of Triphala-derived polyphenols demonstrated strong binding affinities and stability in molecular docking and dynamics simulations, highlighting polyphenols such as chebulagic acid and punicalagin as promising drug-like agents [53]. A GC/MS-assisted metabolomics study on Triphala identified 84 metabolites, with key phenolic compounds such as gallic acid, benzene-1,2,4-triol, and 3,4-dihydroxybenzoic acid driving its antioxidant, anti-malarial, and hepatoprotective properties; notably, the 50% methanolic extract and benzene-1,2,4-triol exhibited significant hepatoprotection and potent β-glucuronidase inhibition, thereby underscoring Triphala’s ethnopharmacological relevance and therapeutic potential in oxidative stress and liver protection [54]. Collectively, these findings highlight Triphala’s chemical diversity and support its potential as a safe, multi-target phytotherapeutic agent. ADME predictions showed strong intestinal absorption and low metabolic liabilities for most Triphala compounds, while fatty acids were limited by poor solubility and possible CYP1A2 interactions. Toxicity profiling confirmed the safety of phenolics, amino acid derivatives, and furan compounds, with only N-acetylglutamic acid and stearic acid requiring caution due to mutagenic or cardiotoxic alerts [55]. Overall, these results highlight Triphala’s chemical diversity and support its drug-like, pharmacokinetic, and safety potential.

Network pharmacology analysis offers a systems-level view of how Triphala interacts with biological pathways related to oxidative stress-induced hemolysis and G6PD deficiency, supporting its role as a multi-target phytotherapeutic agent. The Venn analysis identified 42 overlapping targets between Triphala-associated compounds and disease-related pathways, consistent with the concept that herbal formulations act through network-based mechanisms rather than single targets [32]. The degree analysis ranked PPARG, PTGS2, EGFR, MMP9, TLR4, ACE, REN, PPARA, SERPINE1, and MMP2 as the top hub proteins, indicating that Triphala-derived compounds may protect against hemolysis by modulating key regulators of oxidative stress, inflammation, and metabolic signaling, thereby supporting RBC survival under stress conditions. PPARG, a nuclear receptor regulating lipid metabolism and anti-inflammatory responses, may indirectly support RBC stability by reducing systemic oxidative stress, although it is not expressed in mature erythrocytes [56]. Similarly, EGFR, a receptor tyrosine kinase involved in cellular stress and inflammatory signaling, may affect the oxidative environment around RBCs rather than acting directly within them [57]. MMP9, an extracellular matrix-remodeling protease, is associated with inflammation and oxidative stress and may contribute to a pro-oxidative milieu during hemolysis [58]. From an immunological perspective, TLR4 and PTGS2 are particularly relevant. TLR4, a key pattern recognition receptor, drives innate immune activation and can be triggered by damage-associated molecules released during RBC injury, thereby amplifying inflammatory and oxidative responses. PTGS2 (COX-2), an inducible enzyme, is upregulated during inflammation and contributes to prostaglandin synthesis and oxidative stress [59], both TLR4 and PTGS2 are not expressed in mature RBCs but are activated in surrounding immune and endothelial cells, indicating their roles in hemolysis are indirect and dependent on microenvironment. Together, these findings suggest that Triphala may exert its effects through coordinated modulation of redox-inflammatory networks, rather than acting on RBCs alone. Its polyphenolic constituents may influence key signaling pathways associated with oxidative stress, immune activation, and extracellular remodeling, thereby shaping the systemic environment that contributes to RBC damage. While these interactions are still predictive, they provide a plausible mechanism explaining the link. The modulation of these pathways suggests that Triphala may not only act as an antioxidant but also influence immune-redox crosstalk, which is increasingly recognized as a critical factor in oxidative tissue injury, including hemolysis [51]. In addition, PPARG and PPARA are involved in lipid metabolism and anti-inflammatory signaling, linking metabolic regulation to oxidative stress control. GO Cellular Component analysis also showed enrichment of terms, for example extracellular vesicles, exosomes, and membrane microdomains. This is notable because extracellular vehicles (EVs) play key roles in intercellular communication during oxidative stress and inflammation. In the context of RBC biology, oxidative stress can induce the release of RBC-derived EVs (microvesicles), which contribute to membrane remodeling, removal of damaged components, and modulation of immune responses [50]. Triphala’s association with extracellular vesicle (EV) components suggests it may influence EV formation, cargo, and signaling, thereby modulating oxidative stress and immune responses. This is particularly important in hemolytic conditions, where EVs act as both biomarkers and mediators of pathology. Supporting evidence from GO and KEGG analyses shows involvement in oxidative stress, apoptosis, HIF-1, FoxO, and AGE-RAGE signaling, all interconnected with inflammatory and stress-adaptation pathways. Overall, Triphala appears to act through coordinated regulation of antioxidant, inflammatory, and intercellular communication networks, with TLR4-mediated inflammation, PPAR signaling, and EV biology emerging as key areas for further study in oxidative hemolysis and G6PD deficiency. Moreover, MMP-2 and MMP-9 are zinc-dependent enzymes that remodel the extracellular matrix and become upregulated during hemolysis, as free hemoglobin and heme stimulate their expression in endothelial and synovial cells [60]. Elevated activity, particularly of MMP-9, drives tissue inflammation and damage, linking these enzymes to pathological remodeling in conditions such as stroke, cardiovascular and kidney disease, and chronic hemoglobinopathies [61]. *SERPINE1* encodes Plasminogen Activator Inhibitor-1 (PAI-1), the primary regulator of fibrinolysis by inhibiting blood clot breakdown. During hemolysis, the release of heme and free iron promotes vascular injury and drives SERPINE1 upregulation, which in turn contributes to tissue damage, inflammation, and fibrosis [62]. REN and ACE are key enzymes in the renin-angiotensin system, regulating blood pressure and vascular function, but both are linked to hemolysis under severe stress. Excessive renin and angiotensin II can damage endothelial cells and promote thrombotic microangiopathy, while acute intravascular hemolysis reduces ACE activity through degradation of angiotensin II, disrupting RAS dynamics and further contributing to RBC injury [63]. Triphala appears to exert multi-target protective effects against oxidative hemolysis and G6PD deficiency by modulating hub proteins, inflammatory signaling, and extracellular vesicle biology, though these computational predictions require cautious interpretation and experimental validation.

Molecular docking analysis supported the network-based findings by showing that kynurenic acid, a Triphala constituent, binds favorably to PTGS2 (COX-2) and ACE, both key regulators of inflammatory, oxidative stress, blood pressure and vascular function pathways. The observed hydrogen-bonding and hydrophobic interactions within the active sites of these proteins suggest a potential modulatory effect on signaling pathways involved in redox imbalance and cellular injury. MD simulations confirmed that kynurenic acid binds stably to ACE, with RMSD plateauing near 2.4 Å, RMSF values mostly below 2 Å, and recurrent hydrogen bonding and electrostatic contacts involving key residues such as ASN85, THR92, ARG124, LYS343, and GLU403, underscoring its conformational stability and modulatory potential. Similarly, the PTGS2–kynurenic acid complex demonstrated backbone stability with RMSD plateauing near 3.0 Å and RMSF values below 2 Å, while interaction analyses revealed sustained hydrogen bonding and electrostatic contacts with residues including ASN43, ASP125, ILE124, ARG469, SER126, GLN372, and LYS532, highlighting dynamic yet stable binding and its potential role in modulating PTGS2 activity. Given the established roles of PTGS2-mediated inflammatory responses and ACE-associated oxidative stress in tissue damage and vascular dysfunction, these interactions may contribute to the antioxidant and anti-hemolytic effects observed experimentally [64,65]. Previously kynurenic acid distribution, synthesis, and absorption have been investigated in plants, and was detected in leaves, flowers, and roots of medicinal herbs such as dandelion (*Taraxacum officinale*), common nettle (*Urtica dioica*), and greater celandine (*Chelidonium majus*) [66]. Kynurenic acid is a metabolite of the oxidative degradation of tryptophan along the kynurenine pathway. It functions as an endogenous antagonist of ionotropic glutamate receptors and the α7 nicotinic acetylcholine receptor, while also acting as an agonist of the G-protein-coupled receptor GPR35, which is predominantly expressed in gastrointestinal tissues [67,68]. Kynurenic acid exhibits anticonvulsant and neuroprotective properties and has been shown to modulate neurotransmission in the brain based on electrophysiological studies [69]. Kynurenic acid exhibits antioxidant, anti-inflammatory, and neuroprotective properties, with established links to neurological disorders and emerging roles in energy homeostasis, immune and digestive regulation, and as a potential biomarker for metabolic and endocrine diseases [70]. Furthermore, Kynurenic acid is a well-established metabolite in tryptophan metabolism, with documented implications in the pathophysiology of neurological disorders [71]. Experimentally found that kynurenic acid markedly suppressed the expression of cyclooxygenase-2 (COX-2), a key pro-inflammatory enzyme, while simultaneously enhancing the anti-inflammatory marker Arginase-1. By reducing COX-2 activity, kynurenic acid mitigated the inflammatory cascade and oxidative stress that typically exacerbate neurodegenerative and metabolic disorders [72]. Kynurenic acid modulates GPR35 and AhR activity, with AhR activation shown to upregulate COX-2 expression and exacerbate oxidative stress in kidney disease models [73]. Conversely, reduced COX-2 activity in AhR-deficient mice highlights kynurenic acid’s potential role in regulating inflammatory and oxidative pathways relevant to CKD progression [74]. Kynurenic acid has been shown to lower blood pressure following intracerebral administration by modulating brain renin–angiotensin system activity [75]. In vitro studies further reveal that angiotensin-converting enzyme (ACE) inhibitors differentially influence kynurenic acid synthesis and kynurenine aminotransferase activity in the rat brain cortex, with lisinopril enhancing kynurenic acid production, perindopril showing no effect, and ramipril reducing kynurenic acid levels [75]. Taken together, these findings support the potential of kynurenic acid, a Triphala constituent, to modulate PTGS2 (COX-2) and ACE pathways involved in inflammation, oxidative stress, and vascular regulation, though in vitro, in vivo and clinical experimental validation is still required to confirm its therapeutic relevance.

Overall, Triphala appears to exert its protective effects through coordinated regulation of antioxidant, inflammatory, and intercellular communication networks, with TLR4-mediated inflammation, PPAR signaling, and extracellular vesicle (EV) biology emerging as key areas for further investigation in oxidative hemolysis and G6PD deficiency. In addition, MMP-2, MMP-9, SERPINE1, REN, and ACE highlight systemic pathways potentially influenced by Triphala constituents, as supported by molecular docking and dynamics analyses. Nevertheless, these interactions remain predictive, as the study is based primarily on in silico analyses without target-specific functional or protein expression validation, underscoring the need for cautious interpretation. Future work will involve cellular assays, protein expression studies, in vivo models and clinical evidence to experimentally validate the prioritized targets and further elucidate the molecular mechanisms underlying Triphala’s protective effects. Together, the network pharmacology and docking results suggest that Triphala regulates multiple interconnected signaling networks involved in redox homeostasis rather than acting solely as a direct antioxidant. Collectively, these findings provide mechanistic insight into its antioxidant and anti-hemolytic properties and support its potential as a multi-target therapeutic candidate for oxidative stress-related hematological disorders, while emphasizing that the predicted interactions require experimental validation to confirm their biological relevance and therapeutic significance.

## 5. Conclusions

H_2_O_2_-induced hemolysis in experimental models. Integrated network pharmacology and molecular docking analyses further identified multiple putative molecular targets and pathways associated with oxidative stress, inflammatory responses, and cellular homeostasis, providing a systems-level framework for understanding its biological activities. The context-dependent effects observed in G6PD-deficient erythrocytes further highlight the importance of cellular redox status in modulating the biological responses to phytochemicals. Integration of network pharmacology and molecular docking identified ACE and PTGS2 as key targets of the Triphala-derived compound. MD simulations confirmed that kynurenic acid binds dynamically yet stably to ACE and PTGS2, with RMSD values plateauing near 2.4 Å and 3.0 Å respectively, RMSF values mostly below 2 Å, and recurrent hydrogen bonding and electrostatic contacts involving key residues such as ASN85, THR92, ARG124, LYS343, GLU403 for ACE and ASN43, ASP125, ILE124, ARG469, SER126, GLN372, LYS532 for PTGS2, collectively underscoring its conformational stability, adaptive flexibility, and modulatory potential. Collectively, these findings support the potential of Triphala as a promising natural antioxidant with anti-hemolytic properties. However, the proposed molecular mechanisms remain predictive and require further validation through mechanistic studies and pharmacological investigations. In addition, well-designed pre-clinical and clinical studies are essential to establish its efficacy and safety before considering its therapeutic application in G6PD-deficient patients.

## Figures and Tables

**Table 1:** Compounds Identified in Triphala Decoction by LC-MS and GC-MS base on intensity and purity.

**LC-MS of Triphala**				
**Intensity (I)**	**RT (min)**	**Compound Name**	**Molecular Formular**	**Molecular Weight (g/mol)**
216,844	6.12	Gallic acid	C_7_H_6_O_5_	170.12
153,028	7.84	Pyrogallol	C_6_H_6_O_3_	126.11
146,600	3.24	Pyroglutamic acid	C_5_H_7_NO_3_	129.11
72,650	1.55	Aspartic acid	C_4_H_7_NO_4_	133.10
70,412	1.78	Pipecolic acid	C_6_H_11_NO_2_	129.16
45,254	1.52	Arginine	C_6_H_14_N_4_O_2_	174.20
19,549	8.86	Kynurenic acid	C_10_H_7_NO_3_	189.17
17,200	12.65	Trans-Cinnamic acid	C_9_H_8_O_2_	148.16
9886	3.73	N-Acetylglutamic acid	C_7_H_11_NO_5_	189.17
**GC-MS of Triphala**				
**Intensity (I)**/%Area	**RT (min)**	**Compound Name**	**Molecular Formular**	**Molecular Weight (g/mol)**
56.24	16.065	1,2,3-Benzenetriol (Pyrogallol)	C_6_H_6_O_3_	126.11
9.30	6.184	2H-Pyran-2,6(3H)-dione	C_5_H_4_O_3_	112.08
5.64	8.137	2-Furancarboxylic acid	C_5_H_4_O_3_	112.08
3.26	12.092	5-Hydroxymethylfurfural (HMF)	C_6_H_6_O_3_	126.11
1.81	17.426	Trans-Cinnamic acid	C_9_H_8_O_2_	148.16
1.33	29.284	Hexadecanoic acid	C_16_H_32_O_2_	256.42
0.75	33.063	Octadecanoic acid	C_18_H_36_O_2_	284.48
0.69	3.391	Furfural	C_4_H_3_OCHO	96.08
0.45	3.631	2-Furanmethanol	C_5_H_6_O_2_	98.10

Abbreviations: LC–MS, liquid chromatography–mass spectrometry; GC–MS, gas chromatography–mass spectrometry; RT, retention time; Area (%) = relative peak area contribution within the total ion chromatogram (TIC).

**Table 2:** In silico binding energy (kcal/mol) of Triphala-derived compounds with hub proteins implicated in antioxidant defense against RBC hemolysis.

No.	Compound	Pubchem CID	PPAR (7WOX)	PTGS2 (5KIR)	EGFR (7U9A)	MMP9 (1GKC)	TLR4 (3VQ2)	ACE (1O86)	REN (2V16)	PPARA (7E5I)	SERPINE1 (7AQF)	MMP2 (8H78)
1	Pyrogallol	1057	−4.17	−5.11	−5.28	−5.10	−6.09	−5.59	−4.74	−5.06	−5.02	−4.89
2	Gallic acid	370	−4.75	−5.44	−5.68	−4.64	−4.52	−5.56	−4.83	−5.19	−5.43	−5.01
3	Pyroglutamic acid	7405	−5.65	−5.47	−5.07	−4.19	−4.79	−5.18	−4.64	−4.37	−4.85	−4.76
4	N-Acetylglutamic acid	185	−5.27	−5.57	−5.44	−3.54	−4.96	−5.10	−4.41	−3.54	−4.10	−4.19
5	trans-Cinnamic acid	444539	−5.44	−6.57	−5.59	−5.31	−5.00	−5.69	−5.50	−5.50	−5.91	−4.86
6	5-Hydroxymethylfurfural	237332	−4.83	−5.07	−4.88	−4.77	−5.63	−5.45	−4.79	−4.96	−4.57	−4.52
7	Furfural	7362	−4.01	−4.80	−4.22	−4.10	−4.90	−4.73	−4.35	−3.94	−4.58	−4.00
8	Hexadecanoic acid (palmitic acid)	985	−5.13	−6.12	−5.52	−5.68	−4.20	−4.62	−4.17	−5.03	−5.31	−5.28
9	Octadecanoic acid (stearic acid)	5281	−6.20	−6.63	−6.23	−5.47	−4.62	−5.43	−4.38	−4.64	−4.77	−4.78
10	Pipecolic acid	849	−5.54	−5.33	−5.20	−5.39	−4.86	−5.49	−4.42	−4.72	−5.12	−5.26
11	Kynurenic acid	3845	−6.50	−7.45	−6.05	−6.42	−5.46	−7.12	−5.46	−5.62	−6.05	−5.89
12	2H-Pyran-2,6-dione	574367	−4.68	−5.32	−4.98	−4.92	−5.81	−5.01	−4.66	−4.42	−5.32	−4.68
13	2-Furancarboxylic acid	6919	−4.11	−4.90	−4.33	−3.55	−4.25	−5.23	−4.13	−4.13	−4.03	−4.52
14	5YW501 (Co-crystal inhibitor)		−10.43									
15	RCX601 (Co-crystal inhibitor)			−10.49								
16	M191101 (Co-crystal inhibitor)				−7.55							
17	NHF1448 (Co-crystal inhibitor)					−6.40						
18	LP4300 (Co-crystal inhibitor)						−3.54					
19	LPR702 (Co-crystal inhibitor)							−5.73				
20	C471327 (Co-crystal inhibitor)								−11.16			
21	HW3501 (Co-crystal inhibitor)									−10.22		
22	RV2401 (Co-crystal inhibitor)										−8.16	
23	L2U207 (Co-crystal inhibitor)											−6.29

## Data Availability

The datasets generated and/or analyzed during the current study are available from the corresponding author upon reasonable request.

## Supplementary Materials

The following supporting information can be downloaded at: https://www.mdpi.com/article/10.3390/life16081359/s1, Table S1: Bioactive compounds in Triphala extract; Table S2: Anti-hemolysis test of normal and G6PD deficient RBC in H_2_O_2_-induced conditions; Table S3: In silico prediction of pharmacokinetic and ADMET characteristics of Tripala extract compounds using the pkCSM platform; Table S4: In silico toxicity prediction of Tripala-derived compounds, including mutagenicity (AMES), cardiotoxicity (hERG inhibition), hepatotoxicity, sensitization, and aquatic toxicity parameters, as estimated using the pkCSM platform; Table S5: Forty-two overlapping targets identified as common molecular mediators of Triphala-derived compounds in antioxidant defense against RBC hemolysis in normal and G6PD-deficient models; Table S6: Gene Ontology (GO) enrichment analysis of shared targets of Triphala-derived compounds associated with antioxidant defense against RBC hemolysis in normal and G6PD-deficient models; Table S7: The Kyoto Encyclopedia of Genes and Genomes (KEGG) pathway enrichment analysis of 42 overlapping targets between Triphala-derived compounds associated with antioxidant defense against RBC hemolysis; Table S8: In silico molecular docking of Triphala-derived compounds with the top ten hub target proteins implicated in antioxidant defense against RBC hemolysis.

## Abbreviations

The following abbreviations are used in this manuscript:

AA	Ascorbic acid
ABCB1	ATP Binding Cassette Subfamily B Member 1
ABCC1	ATP Binding Cassette Subfamily C Member 1 (ABCC1 Blood Group)
Abs	Absorbance
ABTS	2,2′-azino-bis(3-ethylbenzothiazoline-6-sulfonic acid)
ACE	Angiotensin I Converting Enzyme
AKR1B1	Aldo-Keto Reductase Family 1 Member B
ALK	ALK Receptor Tyrosine Kinase
ALOX5	Arachidonate 5-Lipoxygenase
ALOX15	Arachidonate 15-Lipoxygenase
AR	Androgen Receptor
BCL2L1	BCL2 Like 1
CA2	Carbonic Anhydrase 2
CYP27B1	Cytochrome P450 family 27 subfamily B member 1
COMT	Catechol-O-Methyltransferase
DPPH	2,2-diphenyl-1-picrylhydrazyl
EDTA	Ethylenediaminetetraacetic acid
EGFR	Epidermal Growth Factor Receptor
FABP1	Fatty Acid Binding Protein 1
F2	Coagulation Factor II, Thrombin
GC-MS	Gas Chromatography-Mass Spectrometry
G6PD	Glucose-6-phosphate dehydrogenase
HBA	Hydrogen bond acceptors
HBD	Hydrogen bond donors
HLA-A	Major Histocompatibility Complex, Class I, A
HMGCR	3-Hydroxy-3-Methylglutaryl-CoA Reductase
HSD11B2	Hydroxysteroid 11-Beta Dehydrogenase 2
H_2_O_2_	Hydrogen peroxide
IDO1	Indoleamine 2,3-Dioxygenase 1
LC-MS	Liquid Chromatography-Mass Spectrometry
MAPK1	Mitogen-Activated Protein Kinase 1
µm	Micrometer
MMP2	Matrix Metallopeptidase 2
MMP9	Matrix Metallopeptidase 9
NOS2 (iNOS)	Nitric Oxide Synthase 2
NOS3 (eNOS)	Nitric Oxide Synthase 3
NPC1L1	NPC1 Like Intracellular Cholesterol Transporter 1
NR1H4 (FXR)	Nuclear Receptor Subfamily 1 Group H Member 4
PPARA	Peroxisome Proliferator Activated Receptor Alpha
PPARG	Peroxisome Proliferator Activated Receptor Gamma
PLA2G2A	Phospholipase A2 Group IIA
PLG	Plasminogen
PRKCA (PKCα)	Protein Kinase C Alpha
PTGS2 (COX-2)	Prostaglandin-Endoperoxide Synthase 2
PTPRC	Protein Tyrosine Phosphatase Receptor Type C
RBC	Red blood cell
REN	Renin
RT	Retention time
SCD	Stearoyl-CoA Desaturase
SERPINE1	Serpin Family E Member 1
SHBG	Sex Hormone Binding Globulin
SLC1A2	Solute Carrier Family 1 Member 2
TERT	Telomerase Reverse Transcriptase
TLR4	Toll Like Receptor 4
TP	Triphala
TTR	Transthyretin
