# Supplementary material for: Integrated Phytochemical, Network Pharmacology, and Molecular Docking Analyses of Triphala Extract Reveal Protective Effects Against H2O2-Induced Oxidative Hemolysis in G6PD-Deficient Erythrocytes
Source: Life (Basel). 2026 Aug 19;16(8):1359. doi: 10.3390/life16081359 (PMC13514139; doi:10.3390/life16081359)
Supplement: Supplementary file 1 [file life-16-01359-s001.zip › life-4417214-supplementary.pdf]

## Supplement data

**Supplement Table S1**. Bioactive compounds in Triphala extract.

| LCMS of Triphala | | | | | |
|---|---|---|---|---|---|
| **Group** | **Intensity (I)** | **RT (min)** | **Compound Name** | **Molecular Formular** | **Molecular Weight (g/mol)** |
| **Phenolic** | | | | | |
| | 246522 | 3.09 | 4-Nitrophenol | $C_6H_5NO_3$ | 139.11 |
| | 216844 | 6.12 | Gallic acid | $C_7H_6O_5$ | 170.12 |
| | 153028 | 7.84 | Pyrogallol | $C_6H_6O_3$ | 126.11 |
| | 31516 | 1.74 | Quinic acid | $C_7H_{12}O_6$ | 192.17 |
| | 20112 | 4.21 | Homogentisic acid | $C_8H_8O_4$ | 168.15 |
| | 17200 | 12.65 | Trans-Cinnamic acid | $C_9H_8O_2$ | 148.16 |
| | 11874 | 11.58 | 4-Hydroxy-3-methoxycinnamaldehyde | $C_{10}H_{10}O_3$ | 178.18 |
| | 6748 | 10.69 | p-Coumaraldehyde | $C_9H_8O_2$ | 148.16 |
| **Flavonoid** | | | | | |
| | 5726 | 2.89 | 4-Methoxychalcone | $C_{16}H_{14}O_2$ | 238.28 |
| **Terpenoid** | | | | | |
| | 152542 | 2.24 | Cyclofenchene | $C_{10}H_{16}$ | 136.24 |
| | 152542 | 2.24 | Carene | $C_{10}H_{16}$ | 136.24 |
| | 76852 | 8.18 | Camphor | $C_{10}H_{16}O$ | 152.23 |
| | 35446 | 12.08 | Limonene | $C_{10}H_{16}$ | 136.24 |
| | 33664 | 0.66 | Alpha-Terpinene | $C_{10}H_{16}$ | 136.24 |
| | 33664 | 0.66 | Borneol | $C_{10}H_{18}O$ | 154.25 |
| | 33200 | 5.47 | Beta-Phellandrene | $C_{10}H_{16}$ | 136.24 |
| | 31038 | 2.69 | Beta-Pinene | $C_{10}H_{16}$ | 136.24 |
| | 31038 | 2.69 | Alpha-Pinene | $C_{10}H_{16}$ | 136.24 |
| | 25718 | 0.66 | (1S,4R)-p-Mentha-2,8-dien-1-ol | $C_{10}H_{16}O$ | 152.23 |
| | 25678 | 4.85 | Dihydrocarvone | $C_{10}H_{16}O$ | 152.23 |

| GCMS of Triphala | | | | | |
|---|---|---|---|---|---|
| **Group** | **Intensity (I)**/%Area | **RT (min)** | **Compound Name** | **Molecular Formular** | **Molecular Weight (g/mol)** |
| **Phenolic** | 56.24 | 16.065 | 1,2,3-Benzenetriol (Pyrogallol) | $C_6H_6O_3$ | 126.11 |
| | 2.82 | 9.916 | 2,3-Dihydro-3,5-dihydroxy-6-methyl-4H-pyran-4-one | $C_6H_8O_4$ | 144.13 |
| | 1.81 | 17.426 | trans-Cinnamic acid | $C_9H_8O_2$ | 148.16 |
| | 1.75 | 19.350 | 2,4-Di-tert-butylphenol | $C_{14}H_{22}O$ | 206.32 |

**Supplement Table S2**. Anti-hemolysis test of normal and G6PD-deficient RBCs in $H_2O_2$-induced condition.

| Extracts | %Hemolysis | |
|---|---|---|
| | **Normal** | **G6PD deficiency** |
| Ascorbic acid 1000 (µg/mL) | 83.90 ± 3.54 (*p*<0.001) | 388.24 ± 103.68 (*p*<0.05) |
| Triphala 1,000 (µg/mL) | 176.12 ± 6.97 (*p*<0.001) | 78.46 ± 11.74 (*p*<0.01) |
| Triphala 25 (µg/mL) | 63.91 ± 3.45 (*p*<0.001) | 45.5 ± 4.01 (*p*<0.005) |

Data are presented as mean ± SD. Statistical analysis was performed by comparing each treatment group with the $H_2O_2$-treated control group.

**Supplement Table S3.** In silico assessment of physicochemical parameters and drug-likeness properties of Triphala extract compounds using the SwissADME platform.

| Compounds from Triphala extracts | | | | Physiochemical properties | | | | | Drug Likeness | | | | | PAINS |
|---|---|---|---|---|---|---|---|---|---|---|---|---|---|---|
| No | Name | Pubchem CID | Formula | MW <500g/mol | HBD <5 | HBA <10 | PSA 140 Å$^2$ | logP < 5 | Lipinski | Ghose | Veber | Egan | Muegge | |
| 1 | Pyrogallol | 1057 | $C_6H_3(OH)_3$ | 126.11 | 3 | 3 | 60.69 | 0.58 | Yes | No | Yes | Yes | No | 1 alert |
| 2 | Gallic acid | 370 | $C_7H_6O_5$ | 170.12 | 4 | 5 | 97.99 | 0.21 | Yes | No | Yes | Yes | No | 1 alert |
| 3 | Pyroglutamic acid | 7405 | $C_5H_7NO_3$ | 129.11 | 2 | 3 | 66.40 | -0.46 | Yes | No | Yes | Yes | No | 0 alert |
| 4 | N-Acetylglutamic acid | 185 | $C_7H_{11}NO_5$ | 189.17 | 3 | 5 | 103.70 | -0.69 | Yes | No | Yes | Yes | No | 0 alert |
| 5 | trans-Cinnamic acid | 444539 | $C_9H_8O_2$ | 148.16 | 2 | 1 | 37.30 | 1.79 | Yes | No | Yes | Yes | No | 0 alert |
| 6 | 5-Hydroxymethylfurfural | 237332 | $C_6H_6O_3$ | 126.11 | 1 | 3 | 50.44 | 0.19 | Yes | No | Yes | Yes | No | 0 alert |
| 7 | Furfural | 7362 | $C_4H_3OCHO$ | 96.08 | 0 | 2 | 30.21 | 0.69 | Yes | No | Yes | Yes | No | 0 alert |
| 8 | Hexadecanoic acid (palmitic acid) | 985 | $C_{16}H_{32}O_2$ | 256.42 | 1 | 2 | 37.30 | 5.20 | Yes | Yes | No | Yes | No | 0 alert |
| 9 | Octadecanoic acid (stearic acid) | 5281 | $C_{18}H_{36}O_2$ | 284.48 | 1 | 2 | 37.3 | 5.93 | Yes | No | No | No | No | 0 alert |
| 10 | Pipecolic acid | 849 | $C_6H_{11}NO_2$ | 129.16 | 2 | 3 | 49.33 | -0.61 | Yes | No | Yes | Yes | No | 0 alert |
| 11 | Kynurenic acid | 3845 | $C_{10}H_7NO_3$ | 189.17 | 2 | 3 | 70.16 | 1.20 | Yes | Yes | Yes | Yes | No | 0 alert |
| 12 | 2H-Pyran-2,6-dione | 574367 | $C_5H_4O_3$ | 112.08 | 0 | 3 | 43.37 | 0.41 | Yes | No | Yes | Yes | No | 0 alert |
| 13 | 2-Furancarboxylic acid | 6919 | $C_5H_4O_3$ | 112.08 | 1 | 3 | 50.44 | 0.56 | Yes | No | Yes | Yes | No | 0 alert |

**Supplement Table S4**. In silico prediction of pharmacokinetic and ADMET characteristics of Triphala extract compounds using the pkCSM platform.

| Compounds from Triphala | | Absorption | | | | | | | Distribution | | | | Metabolism | | | | | | | Excretion | |
|---|---|---|---|---|---|---|---|---|---|---|---|---|---|---|---|---|---|---|---|---|---|
| **No** | **Name** | Water solubility | Caco2 permeability | Intestinal absorption (human) | Skin Permeability | P-glycoprotein substrate | P-glycoprotein I inhibitor | P-glycoprotein II inhibitor | VDss (human) | Fraction unbound (human) | BBB permeability | CNS permeability | CYP2D6 substrate | CYP3A4 substrate | CYP1A2 inhibitior | CYP2C19 inhibitior | CYP2C9 inhibitior | CYP2D6 inhibitior | CYP3A4 inhibitior | Total Clearance | Renal OCT2 substrate |
| 1 | Pyrogallol | -0.094 | 1.165 | 65.746 | -3.370 | Yes | No | No | -0.41 | 0.526 | -0.335 | -2.452 | No | No | No | No | No | No | No | 0.132 | No |
| 2 | Gallic acid | -0.723 | -0.467 | 50.311 | -3.084 | Yes | No | No | -1.078 | 0.565 | -0.930 | -2.816 | No | No | No | No | No | No | No | 0.550 | No |
| 3 | Pyroglutamic acid | 0.177 | 0.148 | 92.50 | -3.148 | Yes | No | No | -0.549 | 0.720 | -0.341 | -3.012 | No | No | No | No | No | No | No | 0.614 | No |
| 4 | N-Acetylglutamic acid | -0.763 | -0.326 | 25.325 | -2.749 | Yes | No | No | -1.210 | 0.699 | -1.055 | -3.523 | No | No | No | No | No | No | No | 0.962 | No |
| 5 | trans-Cinnamic acid | -2.08 | 1.449 | 96.809 | -2.538 | Yes | No | No | -0.565 | 0.395 | 0.256 | -1.443 | No | No | Yes | No | No | No | No | 0.797 | No |
| 6 | 5-Hydroxymethylfurfural | 0.025 | 1.171 | 96.395 | -3.02 | Yes | No | No | -0.206 | 0.621 | -0.237 | -2.643 | No | No | No | No | No | No | No | 0.635 | No |
| 7 | Furfural | -0.500 | 1.291 | 100.00 | -2.348 | Yes | No | No | -0.103 | 0.579 | -0.129 | -2.402 | No | No | No | No | No | No | No | 0.610 | No |
| 8 | Hexadecanoic acid (palmitic acid) | -5.702 | 1.476 | 91.551 | -2.593 | Yes | No | No | 0.159 | 0.186 | 0.028 | -1.307 | No | No | Yes | No | No | No | No | 1.565 | No |
| 9 | Octadecanoic acid (stearic acid) | -6.202 | 1.486 | 90.713 | -2.661 | Yes | No | No | 0.276 | 0.128 | -0.003 | -1.158 | No | No | Yes | No | No | No | No | 1.639 | No |
| 10 | Pipecolic acid | 0.152 | 1.136 | 95.044 | -3.033 | Yes | No | No | 0.161 | 0.761 | -0.240 | -0.240 | No | No | No | No | No | No | No | 0.612 | No |
| 11 | Kynurenic acid | -1.725 | 0.13 | 69.473 | -2.93 | Yes | No | No | -0.868 | 0.437 | -0.029 | -2.387 | No | No | Yes | No | No | No | No | 0.752 | No |
| 12 | 2H-Pyran-2,6-dione | 0.261 | 1.175 | 100.00 | -3.046 | Yes | No | No | -3.046 | 0.654 | -0.237 | -2.517 | No | No | No | No | No | No | No | 0.715 | No |
| 13 | 2-Furancarboxylic acid | -0.496 | 1.170 | 96.397 | -2.875 | Yes | No | No | -0.640 | 0.609 | -0.279 | -2.472 | No | No | No | No | No | No | No | 0.662 | No |

**Supplement Table S5.** In silico toxicity prediction of Triphala-derived compounds, including mutagenicity (AMES), cardiotoxicity (hERG inhibition), hepatotoxicity, sensitization, and aquatic toxicity parameters, as estimated using the pkCSM platform.

| **Compounds from Triphala extract** | | **Toxicity** | | | | | | | | | |
|---|---|---|---|---|---|---|---|---|---|---|---|
| **No** | **Name** | AMES toxicity | Max. tolerated dose (human) | hERG I inhibitor | hERG II inhibitor | Oral Rat Acute Toxicity (LD50) | Oral Rat Chronic Toxicity (LOAEL) | Hepatotoxicity | Skin Sensitisation | T.Pyriformis toxicity | Minnow toxicity |
| 1 | Pyrogallol | No | 1.770 | No | No | 1.959 | 2.936 | No | No | -0.543 | 2.586 |
| 2 | Gallic acid | No | 1.404 | No | No | 1.872 | 1.499 | No | No | -0.071 | 2.918 |
| 3 | Pyroglutamic acid | No | 1.076 | No | No | 2.082 | 2.431 | No | No | -0.974 | 3.292 |
| 4 | N-Acetylglutamic acid | Yes | 0.779 | No | No | 1.761 | 1.132 | No | No | 0.07 | 3.000 |
| 5 | trans-Cinnamic acid | No | 1.566 | No | No | 2.001 | 2.726 | No | Yes | 0.437 | 1.393 |
| 6 | 5-Hydroxymethylfurfural | No | 1.358 | No | No | 2.338 | 2.411 | No | No | -1.432 | 2.992 |
| 7 | Furfural | No | 1.422 | No | No | 2.461 | 1.584 | No | Yes | -1.207 | 2.662 |
| 8 | Hexadecanoic acid (palmitic acid) | No | 1.247 | No | No | 1.916 | 3.123 | No | Yes | 1.744 | -1.733 |
| 9 | Octadecanoic acid (stearic acid) | No | 1.222 | No | Yes | 1.861 | 3.272 | No | Yes | 1.537 | -2.393 |
| 10 | Pipecolic acid | No | 0.971 | No | No | 2.286 | 2.315 | No | No | -0.815 | 3.002 |
| 11 | Kynurenic acid | No | 1.219 | No | No | 2.265 | 2.745 | No | No | 0.568 | 2.495 |
| 12 | 2H-Pyran-2,6-dione | No | 1.219 | No | No | 2.033 | 2.316 | No | Yes | -1.469 | 2.703 |
| 13 | 2-Furancarboxylic acid | No | 1.297 | No | No | 2.468 | 2.428 | No | No | -0.896 | 2.961 |

**Supplement Table S6.** Forty-two overlapping targets identified as common molecular mediators of Triphala-derived compounds in antioxidant defense against RBC hemolysis in normal and G6PD-deficient models.

| Category | Proteins | Full name | Role/Mechanism |
|---|---|---|---|
| **Core RBC Antioxidant Defense** | G6PD | Glucose-6-Phosphate Dehydrogenase | Directly neutralize ROS or regulate redox balance inside RBCs. G6PD supplies NADPH for glutathione recycling; NOS enzymes modulate NO/ROS; PRKCA regulates NADPH oxidase; MAPK1 controls eryptosis; ABCC1/ABCB1 export glutathione conjugates; CA2 buffers pH. |
| | NOS3 (eNOS) | Nitric Oxide Synthase 3 | |
| | NOS2 (iNOS) | Nitric Oxide Synthase 2 | |
| | PRKCA (PKCα) | Protein Kinase C Alpha | |
| | MAPK1 | Mitogen-Activated Protein Kinase 1 | |
| | ABCC1 | ATP Binding Cassette Subfamily C Member 1 (ABCC1 Blood Group) | |
| | ABCB1 | ATP Binding Cassette Subfamily B Member 1 | |
| | CA2 | Carbonic Anhydrase 2 | |
| **Indirect/Systemic Modulators** | ACE | Angiotensin I-Converting Enzyme | Influence systemic oxidative stress, vascular tone, metabolism, and immune signaling. Not RBC-intrinsic but can alter oxidative environment. |
| | REN | Renin | |
| | F2 (thrombin) | Coagulation Factor II, Thrombin | |
| | PLG | Plasminogen | |
| | EGFR | Epidermal Growth Factor Receptor | |
| | ALK | ALK Receptor Tyrosine Kinase | |
| | PTPRC | Protein Tyrosine Phosphatase Receptor Type C | |
| | SERPINE1 | Serpin Family E Member 1 | |
| | COMT | Catechol-O-Methyltransferase | |
| | HSD11B2 | Hydroxysteroid 11-Beta Dehydrogenase 2 | |
| | NPC1L1 | NPC1 Like Intracellular Cholesterol Transporter 1 | |
| | HMGCR | 3-Hydroxy-3-Methylglutaryl-CoA Reductase | |
| | AR | Androgen Receptor | |
| | CYP27B1 | Cytochrome P450 family 27 subfamily B member 1 | |
| | SHBG | Sex Hormone Binding Globulin | |
| | TERT | Telomerase Reverse Transcriptase | |
| **Inflammatory & Oxidative Mediators** | PTGS2 (COX-2) | Prostaglandin-Endoperoxide Synthase 2 | Amplify oxidative damage through prostaglandin synthesis, lipid peroxidation, immune activation, and aldehyde detoxification. Indirect contributors to RBC stress. |
| | ALOX5 | Arachidonate 5-Lipoxygenase | |
| | ALOX15 | Arachidonate 15-Lipoxygenase | |

| Category | Proteins | Full name | Role/Mechanism |
|---|---|---|---|
| | PLA2G2A | Phospholipase A2 Group IIA | |
| | MMP9 | Matrix Metallopeptidase 9 | |
| | MMP2 | Matrix Metallopeptidase 2 | |
| | TLR4 | Toll Like Receptor 4 | |
| | HLA-A | Major Histocompatibility Complex, Class I, A | |
| | IDO1 | Indoleamine 2,3-Dioxygenase 1 | |
| | AKR1B1 | Aldo-Keto Reductase Family 1 Member B | |
| **Metabolic & Lipid Homeostasis** | PPARA | Peroxisome Proliferator Activated Receptor Alpha | Regulate lipid metabolism, desaturation, transport, and antioxidant gene expression. Indirectly influences susceptibility to oxidative damage. |
| | PPARG | Peroxisome Proliferator Activated Receptor Gamma | |
| | FABP1 | Fatty Acid Binding Protein 1 | |
| | SCD | Stearoyl-CoA Desaturase | |
| | TTR | Transthyretin | |
| **Others** | BCL2L1 | BCL2 Like 1 | BCL2L1 functions as an anti-apoptotic regulator, but it is not directly linked to RBC antioxidant defense. SLC1A2 is a glutamate transporter with CNS relevance rather than RBC specificity, while NR1H4 (FXR) is a nuclear receptor involved in bile acid–mediated systemic metabolic regulation. |
| | SLC1A2 | Solute Carrier Family 1 Member 2 | |
| | NR1H4 (FXR) | Nuclear Receptor Subfamily 1 Group H Member 4 | |

**Supplement Table S7.** Gene Ontology (GO) enrichment analysis of shared targets of Triphala-derived compounds associated with antioxidant defense against RBC hemolysis in normal and G6PD-deficient models.

| Enrichment FDR | nGenes | Pathway Genes | Fold Enrichment | Pathway |
|---|---|---|---|---|
| **GO Biological Process** | | | | |
| $2.51 \times 10^{-17}$ | 26 | 1752 | 8.935743 | GO:1901700 response to oxygen-containing compound |
| $3.13 \times 10^{-14}$ | 31 | 4103 | 4.549373 | GO:0065008 reg. of biological quality |
| $1.30 \times 10^{-13}$ | 19 | 980 | 11.67398 | GO:0033993 response to lipid |
| $6.22 \times 10^{-13}$ | 20 | 1272 | 9.467478 | GO:1901701 cellular response to oxygen-containing compound |
| $1.36 \times 10^{-12}$ | 30 | 4424 | 4.083171 | GO:0006950 response to stress |
| $1.36 \times 10^{-12}$ | 22 | 1810 | 7.318726 | GO:0010941 reg. of cell death |
| $1.36 \times 10^{-12}$ | 31 | 4821 | 3.871827 | GO:0042221 response to chemical |
| $3.18 \times 10^{-12}$ | 26 | 3073 | 5.094507 | GO:0009605 response to external stimulus |
| $1.16 \times 10^{-11}$ | 23 | 2320 | 5.969408 | GO:0008219 cell death |
| $1.16 \times 10^{-11}$ | 26 | 3269 | 4.789055 | GO:0010033 response to organic substance |
| $1.16 \times 10^{-11}$ | 25 | 2945 | 5.111474 | GO:0032879 reg. of localization |
| $1.24 \times 10^{-11}$ | 26 | 3300 | 4.744067 | GO:0070887 cellular response to chemical stimulus |
| $1.29 \times 10^{-11}$ | 20 | 1594 | 7.554976 | GO:0042981 reg. of apoptotic proc. |
| $1.63 \times 10^{-11}$ | 18 | 1194 | 9.07736 | GO:0009628 response to abiotic stimulus |
| $1.63 \times 10^{-11}$ | 22 | 2127 | 6.227971 | GO:0012501 programmed cell death |
| **GO Cellular Component** | | | | |
| **Enrichment FDR** | **nGenes** | **Pathway Genes** | **Fold Enrichment** | **Pathway** |
| $1.05 \times 10^{-06}$ | 24 | 4466 | 3.235817 | GO:0031982 vesicle |
| $1.49 \times 10^{-05}$ | 16 | 2343 | 4.111867 | GO:0043230 extracellular organelle |
| $1.49 \times 10^{-05}$ | 16 | 2343 | 4.111867 | GO:0065010 extracellular membrane-bounded organelle |
| $1.49 \times 10^{-05}$ | 16 | 2316 | 4.159804 | GO:0070062 extracellular exosome |
| $1.49 \times 10^{-05}$ | 16 | 2342 | 4.113623 | GO:1903561 extracellular vesicle |
| $2.91 \times 10^{-05}$ | 19 | 3577 | 3.198351 | GO:0005615 extracellular space |
| $3.41 \times 10^{-04}$ | 20 | 4673 | 2.577066 | GO:0005576 extracellular region |
| $1.89 \times 10^{-03}$ | 6 | 429 | 8.421421 | GO:0043235 receptor complex |
| $5.27 \times 10^{-03}$ | 7 | 757 | 5.567927 | GO:0098552 side of membrane |

| | | | | |
|---|---|---|---|---|
| $5.49 \times 10^{-03}$ | 5 | 351 | 8.577373 | GO:0045121 membrane raft |
| $5.49 \times 10^{-03}$ | 5 | 352 | 8.553005 | GO:0098857 membrane microdomain |
| $6.57 \times 10^{-03}$ | 5 | 373 | 8.071469 | GO:0031983 vesicle lumen |
| $6.77 \times 10^{-03}$ | 2 | 18 | 66.90351 | GO:0044214 spanning component of plasma membrane |
| $7.23 \times 10^{-03}$ | 3 | 91 | 19.85049 | GO:0005901 caveola |
| $1.12 \times 10^{-02}$ | 5 | 459 | 6.559168 | GO:0009897 external side of plasma membrane |

**GO Molecular Function**

| Enrichment FDR | nGenes | Pathway Genes | Fold Enrichment | Pathway |
|---|---|---|---|---|
| $1.70 \times 10^{-07}$ | 13 | 841 | 9.307623 | GO:0008289 lipid binding |
| $3.41 \times 10^{-06}$ | 5 | 57 | 52.81856 | GO:0004879 nuclear receptor activity |
| $3.41 \times 10^{-06}$ | 6 | 107 | 33.76439 | GO:0005496 steroid binding |
| $3.41 \times 10^{-06}$ | 5 | 57 | 52.81856 | GO:0098531 ligand-activated transcription factor activity |
| $3.83 \times 10^{-06}$ | 13 | 1247 | 6.277234 | GO:0046914 transition metal ion binding |
| $1.04 \times 10^{-05}$ | 6 | 151 | 23.92576 | GO:0043177 organic acid binding |
| $2.21 \times 10^{-05}$ | 6 | 176 | 20.52721 | GO:0031406 carboxylic acid binding |
| $2.84 \times 10^{-05}$ | 10 | 820 | 7.343068 | GO:0016491 oxidoreductase activity |
| $3.69 \times 10^{-05}$ | 3 | 12 | 150.5329 | GO:0032052 bile acid binding |
| $5.43 \times 10^{-05}$ | 5 | 120 | 25.08882 | GO:0001221 transcription coregulator binding |
| $5.43 \times 10^{-05}$ | 4 | 52 | 46.31781 | GO:0050661 NADP binding |
| $5.71 \times 10^{-05}$ | 3 | 15 | 120.4263 | GO:0046965 nuclear retinoid X receptor binding |
| $5.90 \times 10^{-05}$ | 21 | 4647 | 2.721059 | GO:0046872 metal ion binding |
| $5.96 \times 10^{-05}$ | 10 | 947 | 6.358306 | GO:0008270 zinc ion binding |
| $7.08 \times 10^{-05}$ | 21 | 4737 | 2.669361 | GO:0043169 cation binding |

**Supplement Table S8**. The Kyoto Encyclopedia of Genes and Genomes (KEGG) pathway enrichment analysis of 42 overlapping targets between Triphala-derived compounds associated with antioxidant defense against RBC hemolysis.

| Enrichment FDR | nGenes | Pathway Genes | Fold Enrichment | Pathway |
|---|---|---|---|---|
| $3.22 \times 10^{-10}$ | 13 | 530 | 14.76927 | Path:hsa05200 Pathways in cancer |
| $5.40 \times 10^{-08}$ | 7 | 109 | 38.669 | Path:hsa04066 HIF-1 signaling pathway |
| $8.84 \times 10^{-08}$ | 7 | 129 | 32.67381 | Path:hsa04926 Relaxin signaling pathway |
| $8.84 \times 10^{-08}$ | 8 | 203 | 23.72932 | Path:hsa05415 Diabetic cardiomyopathy |
| $2.36 \times 10^{-06}$ | 7 | 214 | 19.69589 | Path:hsa05417 Lipid and atherosclerosis |
| $8.91 \times 10^{-06}$ | 6 | 161 | 22.43969 | Path:hsa05206 MicroRNAs in cancer |
| $1.43 \times 10^{-05}$ | 13 | 1538 | 5.089539 | Path:hsa01100 Metabolic pathways |
| $1.43 \times 10^{-05}$ | 5 | 100 | 30.10658 | Path:hsa04933 AGE-RAGE signaling pathway in diabetic complications |
| $1.43 \times 10^{-05}$ | 5 | 101 | 29.80849 | Path:hsa05142 Chagas disease |
| $1.43 \times 10^{-05}$ | 4 | 41 | 58.74454 | Path:hsa05219 Bladder cancer |
| $1.59 \times 10^{-05}$ | 6 | 197 | 18.33903 | Path:hsa05207 Chemical carcinogenesis-receptor activation |
| $1.69 \times 10^{-05}$ | 6 | 202 | 17.8851 | Path:hsa05205 Proteoglycans in cancer |
| $3.48 \times 10^{-05}$ | 6 | 232 | 15.57237 | Path:hsa05171 Coronavirus disease-COVID-19 |
| $4.15 \times 10^{-05}$ | 4 | 59 | 40.82248 | Path:hsa04370 VEGF signaling pathway |
| $4.46 \times 10^{-05}$ | 5 | 138 | 21.81636 | Path:hsa04915 Estrogen signaling pathway |
